# Increased Abundance of M Cells in the Gut Epithelium Dramatically Enhances Oral Prion Disease Susceptibility

**DOI:** 10.1371/journal.ppat.1006075

**Published:** 2016-12-14

**Authors:** David S. Donaldson, Anuj Sehgal, Daniel Rios, Ifor R. Williams, Neil A. Mabbott

**Affiliations:** 1 The Roslin Institute & Royal (Dick) School of Veterinary Sciences, University of Edinburgh, United Kingdom; 2 Dept. Pathology, Emory University School of Medicine, Atlanta, Georgia, United States of America; Creighton University, UNITED STATES

## Abstract

Many natural prion diseases of humans and animals are considered to be acquired through oral consumption of contaminated food or pasture. Determining the route by which prions establish host infection will identify the important factors that influence oral prion disease susceptibility and to which intervention strategies can be developed. After exposure, the early accumulation and replication of prions within small intestinal Peyer’s patches is essential for the efficient spread of disease to the brain. To replicate within Peyer’s patches, the prions must first cross the gut epithelium. M cells are specialised epithelial cells within the epithelia covering Peyer’s patches that transcytose particulate antigens and microorganisms. M cell-development is dependent upon RANKL-RANK-signalling, and mice in which RANK is deleted only in the gut epithelium completely lack M cells. In the specific absence of M cells in these mice, the accumulation of prions within Peyer’s patches and the spread of disease to the brain was blocked, demonstrating a critical role for M cells in the initial transfer of prions across the gut epithelium in order to establish host infection. Since pathogens, inflammatory stimuli and aging can modify M cell-density in the gut, these factors may also influence oral prion disease susceptibility. Mice were therefore treated with RANKL to enhance M cell density in the gut. We show that prion uptake from the gut lumen was enhanced in RANKL-treated mice, resulting in shortened survival times and increased disease susceptibility, equivalent to a 10-fold higher infectious titre of prions. Together these data demonstrate that M cells are the critical gatekeepers of oral prion infection, whose density in the gut epithelium directly limits or enhances disease susceptibility. Our data suggest that factors which alter M cell-density in the gut epithelium may be important risk factors which influence host susceptibility to orally acquired prion diseases.

## Author Summary

Prion diseases are infectious neurodegenerative disorders that affect humans and animals. Many natural prion diseases are orally acquired through consumption of contaminated food or pasture. An understanding of how prions infect the intestine will help identify factors that influence disease susceptibility and allow the development of new treatments. After oral infection prions first accumulate within the lymphoid tissues that line the intestine (known as Peyer’s patches) before they spread to the brain where they cause neurodegeneration. To do this, the prions must first cross the intestinal epithelium, a single layer of cells that separates the body from the gut contents. M cells are found within the epithelium that covers the Peyer’s patches and are specialised to transport large particles and whole bacteria across the gut epithelium. We show that M cells act as the gatekeepers of oral prion infection. In the absence of M cells, oral prion infection is blocked, whereas an increase in M cells increases the risk of prion infection and shortens the disease duration. Therefore, our data demonstrate that factors such as pathogen infection, inflammation and aging, which alter the abundance of M cells in the intestine, may be important risk factors which influence susceptibility to orally-acquired prion infections.

## Introduction

Prion diseases (transmissible spongiform encephalopathies) are a unique group of subacute neurodegenerative diseases that affect humans and animals. During prion disease, aggregations of PrP^Sc^, an abnormally folded isoform of cellular PrP (PrP^C^), accumulate in affected tissues. Prion infectivity co-purifies with PrP^Sc^ and constitutes the major, if not sole, component of the infectious agent [1–3]. Many natural prion diseases, including natural sheep scrapie, bovine spongiform encephalopathy (BSE), chronic wasting disease in cervids, and variant Creutzfeldt-Jakob disease in humans (vCJD), are acquired peripherally, such as by oral consumption of prion-contaminated food or pasture. The precise mechanism by which orally-acquired prions are propagated from the gut lumen across the epithelium to establish host infection is uncertain. In the U.K. relatively few vCJD cases have fortunately occurred despite widespread dietary exposure to BSE [4], suggesting that the acquisition of prions from the gut lumen may differ between individuals. Further studies are clearly necessary to precisely characterise the cellular route that prions exploit to establish infection after oral exposure, and how alterations to this cellular route, both intrinsic and extrinsic, can affect disease susceptibility. Treatments which prevent the accumulation and replication of prions in host lymphoid tissues can significantly reduce disease susceptibility [5–9]. Therefore, identification of the cellular route by which prions are first transported across the gut epithelium to achieve host infection will identify an important factor which influences oral prion disease susceptibility and to which intervention strategies can be developed.

Following oral exposure the early accumulation and replication of prions upon follicular dendritic cells (FDC) within the gut associated lymphoid tissues (GALT), such as Peyer’s patches of the small intestine, is essential for efficient neuroinvasion [7, 10–13]. FDC are a unique subset of stromal cells resident within the primary B cell follicles and germinal centres of lymphoid tissues [14]. After amplification upon the surface of FDC [15], the prions then infect neighbouring enteric nerves and spread along these to the CNS (a process termed neuroinvasion) where they ultimately cause neurodegeneration and death of the host [16–19].

The follicle-associated epithelia (FAE) which covers the lumenal surfaces of the Peyer’s patches contains a unique population of epithelial cells, termed M cells. These highly phagocytic epithelial cells are specialized for the trans-epithelial transfer of particulate antigens and microorganisms from the gut lumen (termed *transcytosis*) [20], an important initial step in the induction of efficient mucosal immune responses against certain pathogenic bacteria [21, 22] and the commensal bacterial flora [23]. A variety of bacterial and viral pathogens including *Brucella abortus* [24], *Salmonella* Typhimurium [25], *Yersinia enterocolitica* [26], norovirus [27, 28] and reovirus [28] appear to exploit the transcytotic activity of M cells to cross the gut epithelium and infect the host. The food-borne botulinum neurotoxin [29] has also been suggested to exert its toxicity after transcytosis by M cells [29]. Independent studies suggest orally administered prions may similarly be transported by M cells into host tissues [9, 30–32] and that this transport may be important to establish host infection [9]. Other studies have also suggested that prions can be transported across the gut epithelium via enterocytes, independently of M cells [16, 33, 34], however to what extent enterocyte-transported prions contribute to the establishment of host infection has not been assessed.

The differentiation of M cells from uncommitted precursors in the intestinal crypts is critically dependent on stimulation from the cytokine known as RANKL (receptor activator of nuclear factor-κB ligand). This cytokine is expressed by subepithelial stromal cells beneath the FAE in Peyer’s patches, and signals via its receptor RANK (receptor activator of nuclear factor-κB) which is expressed by epithelial cells throughout the intestine [35]. Accordingly, M cell-differentiation is blocked in RANKL-deficient mice or following *in vivo* RANKL-neutralization with anti-RANKL antibody [35]. RANKL stimulation induces a program of gene expression in intestinal epithelial cells which includes the transcription factor SPIB. Expression of SPIB by intestinal epithelial cells is essential for their differentiation and functional maturation into M cells [22, 36, 37]. We have previously reported that the early accumulation of prions upon FDC in Peyer’s patches and subsequent neuroinvasion were blocked in mice in which M cells were transiently depleted by RANKL-neutralization using anti-RANKL antibody [9]. However, since RANKL-RANK signalling has multiple roles in the immune system, a more refined model is required to specifically determine the role of M cells in oral prion disease pathogenesis. In the current study a unique conditional knockout mouse model was used in which RANK expression was specifically deleted only in the intestinal epithelium (RANK^ΔIEC^ mice) [23, 38]. In these mice the complete loss of M cells prevents M cell-mediated antigen uptake from the gut lumen, without altering other RANKL-RANK signalling events required for normal immune development and function [23, 38]. Using these mice our data clearly show that M cells are critically required for the initial trans-epithelial transfer of prions across the gut epithelium into Peyer’s patches in order to establish host infection.

Certain pathogenic bacteria [25, 39] or exposure to inflammatory stimuli such as cholera toxin [40] can significantly increase the density of M cells in the intestine. Inflammation or pathogen infection can also influence prion disease pathogenesis by enhancing the uptake, or expanding the distribution, of prions within the host [11, 41–43]. This raised the hypothesis that exposure to inflammatory stimuli that enhance M cell-density might increase oral prion disease susceptibility by enhancing the uptake of prions from the gut lumen. We show that increased M cell-density at the time of oral exposure dramatically enhanced the uptake of prions from the gut lumen, decreased survival times and increased disease susceptibility by approximately 10-fold. Our data provide a significant advance in our understanding of how prions exploit M cells to initially infect Peyer’s patches and how factors that increase the density of M cells in the gut epithelium, such as concurrent pathogen infection, may have the potential to increase susceptibility to orally-acquired prion infection.

## Results

### RANK-deficiency only in the intestinal epithelium specifically blocks M cell-development

**Fig 1:**
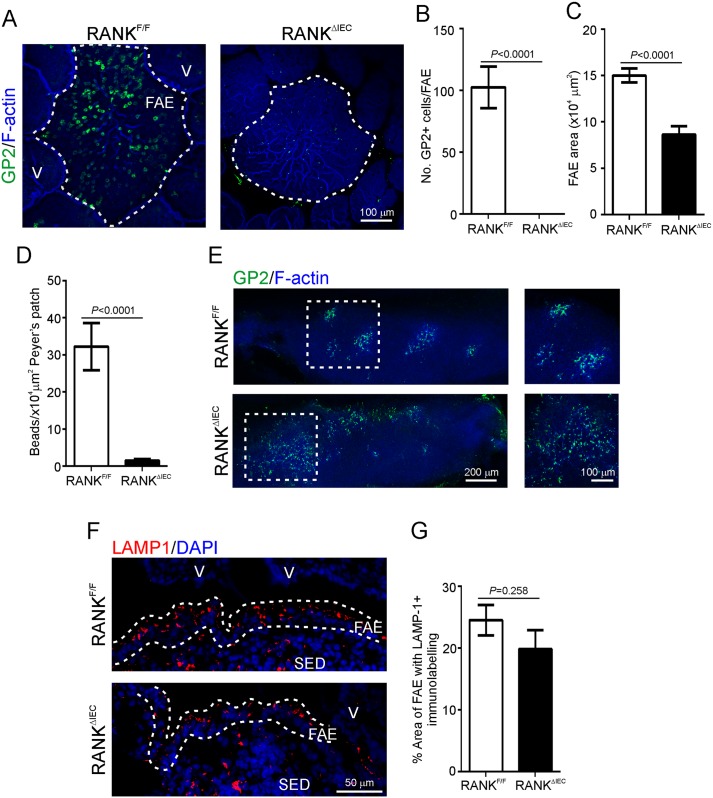
RANK^ΔIEC^ mice specifically lack intestinal M cells. A) Peyer’s patches from RANK^F/F^ and RANK^ΔIEC^ mice were whole-mount immunostained to detect M cells (GP2^+^ cells, green) and F-actin (blue) as a counterstain. The broken line indicates the boundary of the follicle associated epithelium (FAE) overlying the Peyer’s patches. V, villi. This immunohistochemical (IHC) analysis indicated an absence of GP2^+^ M cells in the FAE of RANK^ΔIEC^ mice. B) Morphometric analysis confirmed that the number of GP2^+^ cells/FAE was significantly reduced in RANK^ΔIEC^ mice (*P*<0.0001, Mann-Whitney *U* test). C) The size of the FAE area was also significantly reduced in RANK^ΔIEC^ mice (*P*<0.0001, Student’s *t*-test; data derived from 4 FAE/mouse, *n* = 3–5 mice/group). D) To compare the functional ability of M cells in the FAE of RANK^ΔIEC^ and RANK^F/F^ control mice to transcytose particulate antigens, mice were orally gavaged with 200 nm fluorescent microbeads and 24 h later, the presence of the microbeads in their Peyer’s patches was determined by fluorescence microscopy. The uptake of microbeads into the Peyer’s patches of RANK^ΔIEC^ mice was significantly impaired when compared to RANK^F/F^ mice (*P*<0.0001, Mann-Whitney *U* test; data derived from 21–31 sections of Peyer’s patches/mouse, *n* = 3 mice/group). E) Whole-mount IHC analysis revealed that GP2^+^ M cells (green) were abundant in the nasal associated lymphoid tissues of RANK^F/F^ and RANK^ΔIEC^ mice. F-actin (blue) was used as a counterstain. The boxed area in the left-hand images is shown at higher magnification in the right-hand images. F) IHC analysis was used to compare the presence of large LAMP1^+^ endosomes (red) within enterocytes in FAE of Peyer’s patches from RANK^F/F^ and RANK^ΔIEC^ mice. Sections were counterstained with DAPI (blue) to detect cell nuclei. The broken lines indicate the boundary of the FAE. SED, subepithelial dome. G) Morphometric analysis revealed that the area of the LAMP1^+^ immunostaining in the FAE of RANK^F/F^ and RANK^ΔIEC^ mice was similar (*P* = 0.258, Student’s *t*-test; data derived from 2–8 FAE/mouse, *n* = 3 mice/group), suggesting that the formation of the large LAMP1^+^ endosomes in FAE enterocytes was not influenced by RANK-deficiency in the gut epithelium.

Our previous study showed that oral prion infection was blocked after transient M cell-depletion by treatment with anti-RANKL antibody, implying a functional role for M cells in the trafficking of prions from the lumen into GALT *in vivo* [9]. Although the major phenotype observed in the intestine was a transient loss of mature M cells, RANKL-RANK signalling is also important in immune system and lymphoid tissue development. Therefore, systemic RANKL neutralization by treatment with anti-RANKL antibody could have affected other important cellular processes involved in prion pathogenesis. To exclude these, we used a more refined model of M cell-deficiency, RANK^ΔIEC^ mice [23, 38], to further elucidate the role of M cells in the transport of prions from the intestinal lumen into GALT. These mice are specifically deficient in *Tnfrsf11a* (which encodes RANK) only in *Vil1*-expressing intestinal epithelial cells. As previously published [23], whole-mount immunostaining for the mature M cell marker glycoprotein 2 (GP2; [21, 22]) revealed an absence of GP2^+^ M cells in the FAE of the Peyer’s patches of RANK^ΔIEC^ mice compared to control (RANK^F/F^) mice (Fig 1A & 1B). Coincident with the loss of RANK expression in the gut epithelium was a significant reduction in area of the FAE (Fig 1C).

Assessment of the uptake of fluorescent latex microbeads from the gut lumen into Peyer’s patches is a reliable *in vivo* method to compare the functional ability of M cells to acquire and transcytose particulate antigens. Here, RANK^ΔIEC^ mice and RANK^F/F^ control mice (*n* = 3/group) were orally gavaged with 2x10^11^ 200 nm fluorescent microbeads, and 24 h later the number of microbeads in their Peyer’s patches quantified by fluorescence microscopy. This duration was selected to ensure sufficient time for the beads to transit through the intestine and be transcytosed by M cells in the FAE overlying the Peyer’s patches [13]. Coincident with the absence of mature GP2^+^ M cells, RANK^ΔIEC^ mice had substantially less fluorescent microbeads within the subepithelial dome (SED) regions of their Peyer’s patches when compared to controls (Fig 1D), indicating a dramatic reduction in the ability to sample particulate antigen from the gut lumen.

RANK-dependent GP2^+^ M cells have been described in the epithelium of the nasal associated lymphoid tissue (NALT) [44, 45]. The abundance of GP2^+^ M cells in the NALT was unaffected in RANK^ΔIEC^ mice (Fig 1E), highlighting the intestinal specificity of the model.

In addition to being transported through M cells, prions have also been observed trafficking into Peyer’s patches through the large LAMP1^+^ endosomes of FAE enterocytes [16]. Immunohistochemical (IHC) analysis of LAMP1 expression showed that these endosomes were still present in the FAE of RANK^ΔIEC^ mice (Fig 1F). If the presence of these endosomes in the FAE was dependent on RANKL-RANK signalling, we reasoned that the abundance of LAMP1^+^ immunostaining would be decreased in the FAE of RANK^ΔIEC^ mice. However, morphometric analysis indicated equivalent areas of LAMP1^+^ immunostaining in the FAE of RANK^ΔIEC^ and RANK^F/F^ mice (Fig 1G). These data suggest that the presence of LAMP1^+^ endosomes in the FAE was not RANKL-RANK signalling dependent.

**Fig 2:**
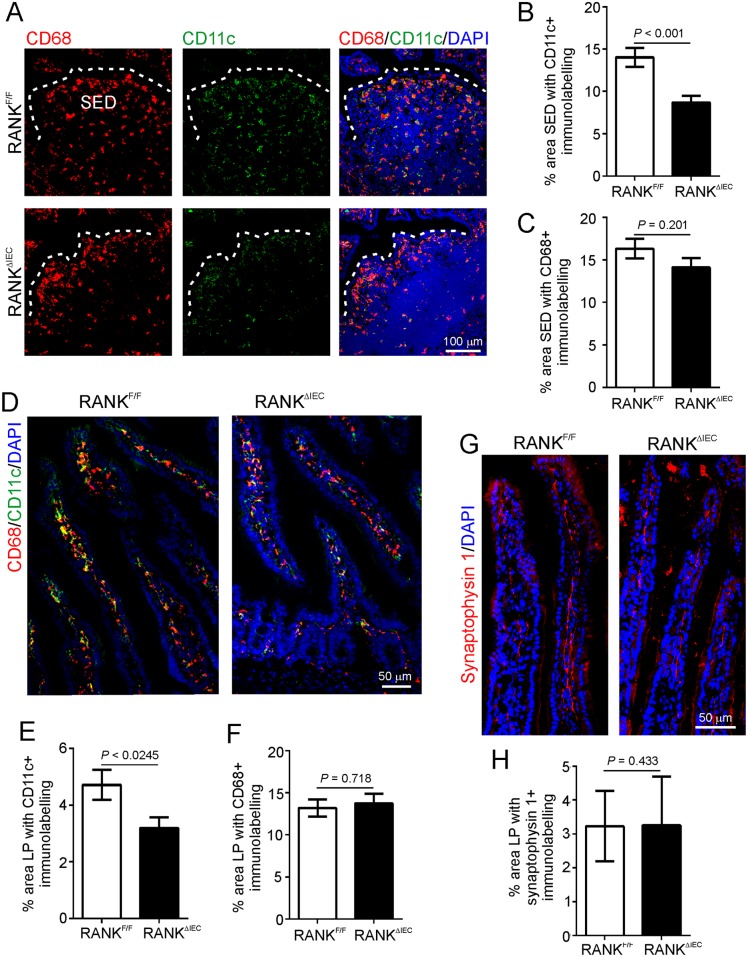
Effect of intestinal epithelial cell-specific RANK-deficiency on mononuclear phagocytes and innervation in the lamina propria. A) Immunohistochemical (IHC) comparison of the distribution of CD11c^+^ (green) and CD68^+^ (red) mononuclear phagocytes (indicative of classical DC and tissue macrophages, respectively) in Peyer’s patches from RANK^F/F^ and RANK^ΔIEC^ mice. Sections were counterstained with DAPI (blue) to detect cell nuclei. Broken line shows the lumenal boundary of the follicle associated epithelium. SED, subepithelial dome. Morphometric analysis revealed that (B) the % area of the SED occupied by CD11c^+^ immunostaining was significantly reduced in RANK^ΔIEC^ mice (*P* < 0.001, Mann-Whitney *U* test), whereas (C) the % area occupied by CD68^+^ immunostaining was similar between each mouse group (*P* = 0.201, Student’s *t*-test; data derived from 1–10 SED/mouse, *n* = 6–7 mice/group). D) IHC comparison of the distribution of CD11c^+^ (green) and CD68^+^ (red) mononuclear phagocytes in the lamina propria (LP) of RANK^F/F^ and RANK^ΔIEC^ mice. Sections were counterstained with DAPI (blue). Morphometric analysis revealed that (E) the % area of the CD11c^+^ immunostaining was also significantly reduced in the LP of RANK^ΔIEC^ mice (*P* < 0.025, Mann-Whitney *U* test), whereas (F) the % area of CD68^+^ immunostaining was similar (*P* = 0.718, Student’s *t*-test; data derived from 1–6 LP areas/mouse, *n* = 7 mice/group). G) Sections of intestine from RANK^F/F^ and RANK^ΔIEC^ mice were immunostained to identify synaptophysin 1 (red) within synaptic vesicles, enabling the enteric innervation in the gut wall to be compared. Sections were counterstained with DAPI (blue). H) Morphometric analysis revealed that the % area of synaptophysin 1^+^ immunostaining within the LP of RANK^F/F^ and RANK^ΔIEC^ mice was similar (*P* = 0.433, Mann-Whitney *U* test; data derived from 2 LP areas/mouse, *n* = 4 mice/group). These data implied that RANK-deficiency in the gut epithelium did not influence the enteric innervation in the gut wall.

Antigens that are transcytosed by M cells are released into their basolateral pockets where they are sampled by lymphocytes and mononuclear phagocytes (MNP; a heterogeneous population of macrophages and classical dendritic cells; DC) [46–48]. The acquisition of prions by MNP such as CD11c^+^ classical DC may mediate their initial transport to FDC [8, 16, 49], and the subsequent transfer of prions from FDC to the peripheral nervous system [50–52]. IHC and morphometric analysis revealed a significant reduction in the % area of CD11c-specific immunostaining in the SED of the Peyer’s patches from RANK^ΔIEC^ mice (Fig 2A & 2B), whereas the % area of CD68-specific immunostaining (indicative of tissue macrophages) was equivalent in RANK^ΔIEC^ and RANK^F/F^ mice (Fig 2A & 2C). Analysis of the intestinal lamina propria (LP) showed a similar trend (Fig 2D–2F).

Following replication upon FDC, the prions subsequently infect enteric nerves (both sympathetic and parasympathetic) to reach the CNS where they ultimately cause neurodegenerative disease [16, 18]. Our IHC analysis of the expression of the neuronal synaptic vesicle marker synaptophysin 1 suggested that the magnitude of the enteric innervation in the LP was similar in the intestines of RANK^ΔIEC^ and RANK^F/F^ mice (Fig 2G & 2H).

Together these data demonstrate that RANK^ΔIEC^ mice represent a refined model in which to study the specific role of M cells in oral prion disease pathogenesis.

### Prion accumulation and dissemination after intra-peritoneal injection is preserved in RANK^ΔIEC^ mice

**Fig 3:**
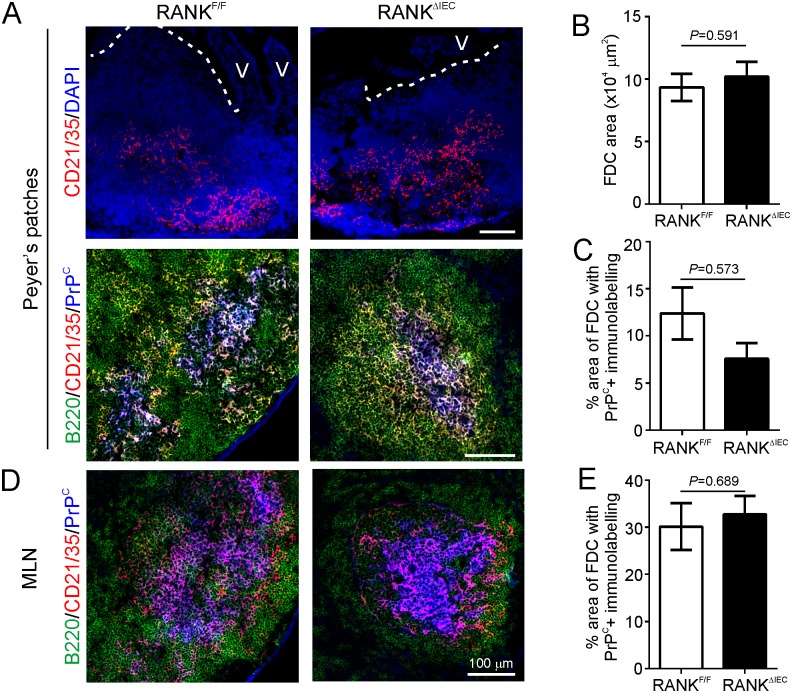
Follicular dendritic cells status in RANK^ΔIEC^ mice. Immunohistochemical (IHC) and morphometric analyses were used to compare follicular dendritic cell (FDC) status in the Peyer’s patches and mesenteric lymph nodes (MLN) of RANK^ΔIEC^ and RANK^F/F^ control mice. A) IHC comparison of CD21/35 (red) and PrP^C^ (blue, lower panels) expression by FDC in the B cell-follicles (B220^+^ cells, green) of Peyer’s patches from RANK^F/F^ and RANK^ΔIEC^ mice. Broken lines show the lumenal surface of the follicle-associated epithelium. V, villi (V). Cell nuclei in the upper panels were counterstained with DAPI (blue). B) Morphometric analysis revealed that the area of the CD21/35^+^ immunostaining in Peyer’s patches from RANK^F/F^ and RANK^ΔIEC^ mice was similar, implying that the FDC networks (CD21/35^+^ cells) in these tissues were of equivalent size (*P* = 0.591, Student’s *t*-test; data derived from 2–9 B cell-follicles/mouse, *n* = 7 mice/group). C) Morphometric analysis suggested that the % area of PrP^C^ immunostaining within the FDC networks was similar in Peyer’s patches from RANK^F/F^ and RANK^ΔIEC^ mice (*P* = 0.573, Mann-Whitney *U* test; data derived from 2–9 B cell-follicles/mouse, *n* = 7 mice/group). D) Sections of MLN from RANK^F/F^ and RANK^ΔIEC^ mice were immunostained to detect B cells (B220, green), FDC (CD21/35^+^ cells, red) and PrP^C^ (blue). E) Morphometric analysis also revealed that the % area of PrP^C^ immunostaining within the FDC networks was similar in the MLN from RANK^F/F^ and RANK^ΔIEC^ mice (*P* = 0.689, Student’s *t*-test; data derived from 3 B cell-follicles/mouse, *n* = 4 mice/group).

The early replication of many prion strains upon FDC within the B cell-follicles of the draining lymphoid tissues is essential for their efficient transmission to the CNS after peripheral exposure [5–7, 15]. FDC in mice characteristically express high levels of CD21/35 (complement receptors 2 & 1, respectively). Our IHC analysis showed that the area of CD21/35-specific immunostaining in Peyer’s patches of 10 wk old RANK^ΔIEC^ and RANK^F/F^ mice was similar (Fig 3A & 3B), suggesting that the size of the FDC networks (CD21/35^+^ cells) in the Peyer’s patches of each mouse strain was equivalent. The replication of prions upon FDC is critically dependent on their expression of PrP^C^ [15, 53, 54]. Morphometric analysis also indicated that the magnitude of the PrP^C^-expression co-localized upon CD21/35^+^ FDC in the Peyer’s patches (Fig 3A & 3C) and mesenteric lymph nodes (MLN) (Fig 3D & 3E) of RANK^ΔIEC^ mice and RANK^F/F^ mice was similar.

**Fig 4:**
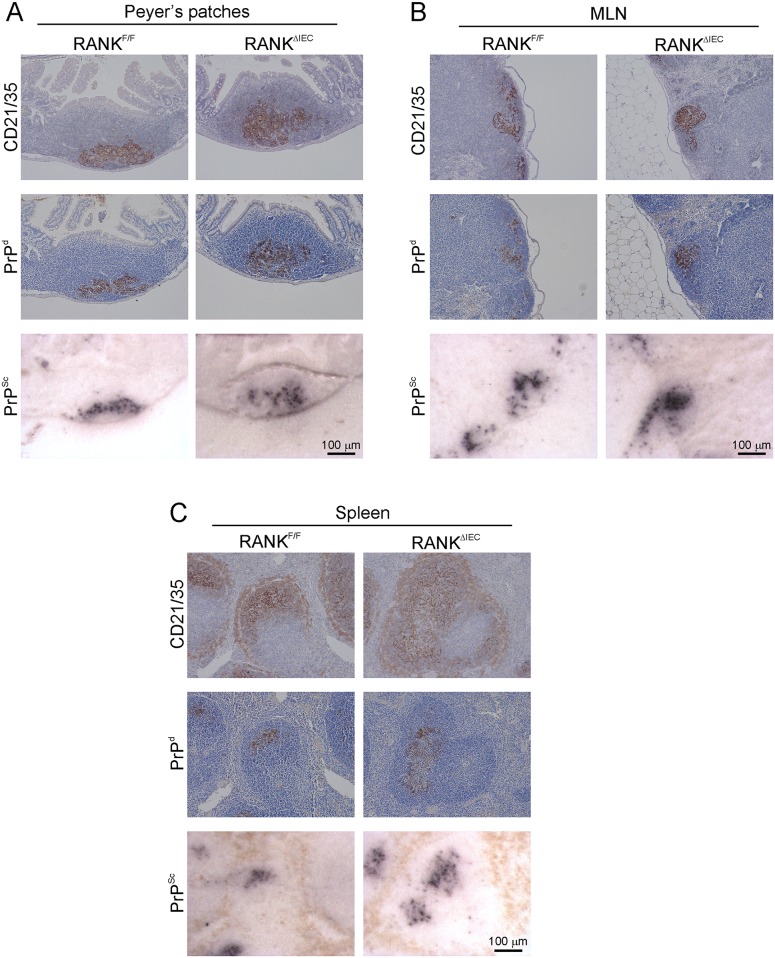
Prion accumulation upon follicular dendritic cells (FDC) after intra-peritoneal injection is preserved in RANK^ΔIEC^ mice. Since prions do not need to cross the gut epithelium to eventually infect Peyer’s patches after injection by the intra-peritoneal route, RANK^ΔIEC^ and RANK^F/F^ (control) were injected with a 1% dose of ME7 scrapie prions via this route and tissues collected at 140 d post-infection, to determine whether the FDC in the lymphoid tissues of RANK^ΔIEC^ mice were capable of accumulating prions. High levels of disease-specific PrP (PrP^d^, brown, middle rows) were detected in association with FDC (CD21/35^+^ cells, brown, upper panels) in the Peyer’s patches (A), mesenteric lymph nodes (MLN, B), and spleens (C) of mice from each group. Sections were counterstained with haematoxylin to detect cell nuclei (blue). Analysis of adjacent sections by PET immunoblot analysis confirmed the presence of prion-specific PK-resistant PrP^Sc^ (blue/black). Images are representative of 4 mice/group.

We next determined whether the FDC in the lymphoid tissues of RANK^ΔIEC^ mice were capable of accumulating prions to a similar extent as those of control mice. After injection by the intra-peritoneal (i.p.) route high levels of prion accumulation and replication are first detected in the spleen within 35 d post infection (dpi) [53]. The prions are then subsequently disseminated around the host via the blood and lymph to most other secondary lymphoid tissues [55]. Furthermore, by 140 dpi the prions are also detectable within Peyer’s patches. Since the prions do not need to cross the gut epithelium to eventually infect the Peyer’s patches after injection by the i.p. route, RANK^ΔIEC^ and RANK^F/F^ were injected with a 1% dose of ME7 scrapie prions via this route and tissues collected at 140 dpi, to determine whether the FDC in the lymphoid tissues of RANK^ΔIEC^ mice were capable of accumulating prions. Prion disease-specific accumulations of PrP (referred to as PrP^d^) were detected by immunostaining for the abnormal aggregates of PrP characteristically present only in affected tissues [6, 9, 11, 13, 53, 56], complimented with paraffin-embedded tissue (PET) blot analysis of adjacent membrane-bound sections to confirm that these aggregates contained relatively proteinase-K (PK)-resistant prion disease-specific PrP^Sc^ [57]. Abundant accumulations of PrP^Sc^ were evident in association with FDC (CD21/35^+^ cells) in the Peyer’s patches, MLN and spleens of RANK^ΔIEC^ and RANK^F/F^ mice (Fig 4A–4C). These data clearly show that the FDC in the Peyer’s patches, MLN and spleen of RANK^ΔIEC^ mice were functionally capable of acquiring and accumulating prions, and that the dissemination of prions between lymphoid tissues was not impaired. Importantly, these data also suggest that the cause of any difference in prion pathogenesis between RANK^ΔIEC^ and RANK^F/F^ mice observed after oral exposure would be restricted to effects on M cells in the gut epithelium.

### RANK^ΔIEC^ mice are resistant to oral prion infection

**Fig 5:**
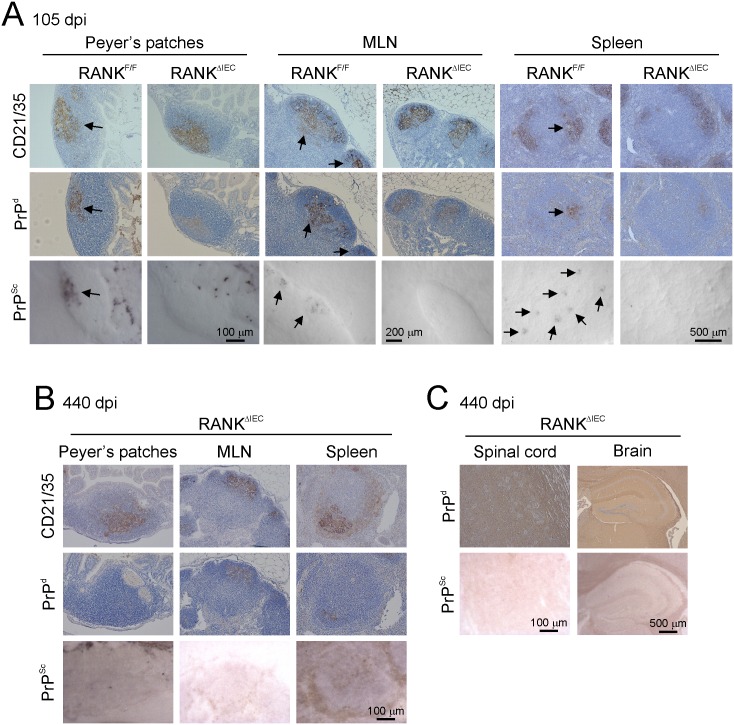
PrP^Sc^ accumulation upon follicular dendritic cells (FDC) after oral prion exposure is blocked in RANK^ΔIEC^ mice. In order to determine the effect of specific M cell-deficiency on oral prion pathogenesis, RANK^ΔIEC^ mice and RANK^F/F^ (control) mice were orally-exposed to a 1% dose of ME7 scrapie prions and tissues collected at intervals afterwards. At 105 d post infection (dpi) high levels of disease-specific PrP (PrP^d^, brown, middle rows) were detected in association with FDC (CD21/35^+^ cells, brown, upper panels) in (A) the Peyer’s patches, (B) mesenteric lymph nodes (MLN), and (C) spleens of RANK^F/F^ control mice. Analysis of adjacent sections by PET immunoblot analysis confirmed the presence of prion-specific PK-resistant PrP^Sc^ (blue/black). Arrows show PrP^d^/PrP^Sc^ accumulation upon FDC networks. In contrast, no PrP^Sc^ was detected in any of the Peyer’s patches, MLN and spleens from orally-exposed RANK^ΔIEC^ mice at (A) 105 dpi or (B) 440 dpi. C) Similarly, no PrP^Sc^ was detected in any of the spinal cords or brains of orally-exposed RANK^ΔIEC^ mice at 440 dpi. Sections were counterstained with haematoxylin to detect cell nuclei (blue). Images are representative of 8 orally-exposed RANK^ΔIEC^ mice.

Within weeks after oral exposure, high levels of ME7 scrapie prions first accumulate upon FDC in the Peyer’s patches and subsequently spread to the MLN and spleen [7–9, 11, 13]. The initial replication of prions upon FDC in the Peyer’s patches is essential for the efficient transmission of disease to the CNS [7, 11, 13]. In order to determine the effect of specific M cell-deficiency on oral prion disease pathogenesis, RANK^ΔIEC^ mice and RANK^F/F^ (control) mice were orally exposed to a moderate dose of ME7 scrapie prions (50 μl of a 1% brain homogenate from a mouse clinically-affected with ME7 scrapie prions; [7, 9, 11, 13, 58]). At intervals after exposure the accumulation of PrP^d^ and PrP^Sc^ in tissues from 4 mice/group were compared by IHC and PET blot analysis, respectively, as above. As anticipated, at 105 dpi, abundant accumulations of PrP^d^ (middle row, brown) and PrP^Sc^ (lower row, black) were detected in association with FDC (CD21/35^+^ cells, upper row, brown) in the Peyer’s patches, MLN and spleen of RANK^F/F^ control mice (Fig 5A). However, no PrP^Sc^ accumulations were detected in the same tissues from RANK^ΔIEC^ mice (Fig 5A). Mice on a C57BL/6 background typically succumb to a moderate dose of ME7 scrapie prions by ~340 d after oral exposure [9, 13]. However, RANK^ΔIEC^ mice (*n* = 8) remained free of the clinical signs of prion disease up to at least 440 dpi, at which point no PrP^d^ or PrP^Sc^ was detected in their Peyer’s patches, MLN, spleen (Fig 5B), spinal cords or brains (Fig 5C) by IHC and PET blot analysis. Together these data clearly show that M cells are essential for the initial uptake of prions from the gut lumen into Peyer’s patches in order to establish host infection, since oral prion disease pathogenesis was blocked in the specific absence of M cells in RANK^ΔIEC^ mice.

### RANKL-treatment promotes M cell development in the FAE and villous epithelium

Certain pathogen infections or inflammatory conditions can enhance M cell-differentiation within the intestine [25, 39, 40]. We therefore reasoned that alterations to M cell-density in the gut epithelium may significantly alter oral prion disease pathogenesis and susceptibility. The density of functionally mature M cells in the intestine can be promoted in mice through exogenous administration of RANKL [22, 35]. Recombinant RANKL was prepared and its ability to stimulate M cell-differentiation was confirmed in *in vitro* intestinal enteroids derived from RANK^ΔIEC^ and RANK^F/F^ mice [23, 36]. As anticipated, RANKL-treatment of enteroids from RANK^F/F^ (control) mice induced robust expression of several M cell-associated genes (*Marcksl1*, *Anxa5*, *Spib*, *Ccl9*, and *Gp2*; [22]) without significantly altering expression of genes associated with other intestinal lineages, including Paneth cells (*Lyz1*, *Lyz2*) and intestinal stem cells (*Lgr5*) (S1 Fig). No induction of expression of M cell-specific genes was observed in RANKL-treated enteroids derived from RANK^ΔIEC^ mice.

**Fig 6:**
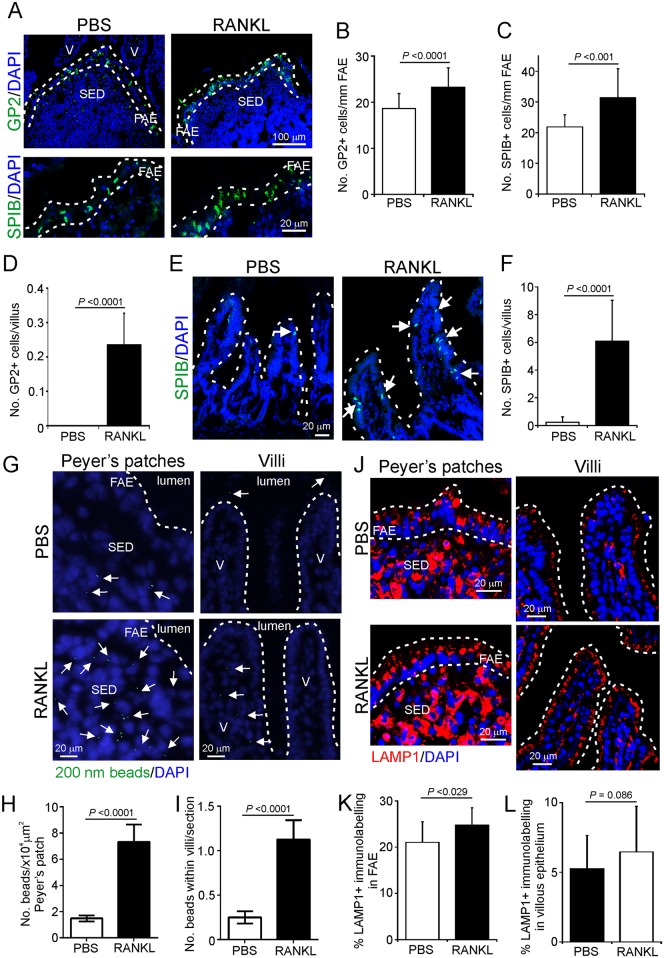
RANKL treatment enhances the density of M cells in the follicle associated epithelium (FAE) of the Peyer’s patches and the villous epithelium of the intestine. The density of functionally mature M cells in the intestine can be promoted in mice through exogenous administration of RANKL. Here, C57BL/6 mice were treated daily with RANKL (or PBS as a control) to induce M cell-differentiation. Peyer’s patches and intestines were collected on d 3 of treatment, coincident with the peak period of induction of M-cell gene expression in the gut epithelium [22, 35]. A-F) Sections of Peyer’s patches and villous epithelium were stained for the M cell markers GP2 and SPIB. A) Representative distribution of GP2^+^ (green, mature M cells, upper-panels) and SPIB^+^ (green, within the nuclei of differentiating and mature M cells, lower panels) M cells in the Peyer’s patches of mice from each group. Broken lines indicate the boundary of the FAE. SED, subepithelial dome; V, villi. Morphometric analysis confirmed a significant increase in the number of (B) GP2^+^ and (C) SPIB^+^ M cells in the Peyer’s patches of RANKL-treated mice (GP2, P<0.0001; SPIB, P<0.001, Student’s *t*-test; 4–5 FAE/mouse, *n* = 4 mice/group). D) Morphometric analysis also revealed a significant increase in the number of GP2^+^ M cells in the villous epithelium after RANKL-treatment (*P*<0.0001, Mann-Whitney *U* test; 5–7 sections/mouse, *n* = 4 mice/group). E) Representative distribution of SPIB^+^ cells (green, arrows) in the villous epithelium of RANKL- and PBS-treated mice. Broken lines indicate the lumenal surface of the gut epithelium. F) Morphometric analysis confirmed a significant increase in the number of SPIB^+^ cells in the villous epithelium after RANKL-treatment (*P*<0.0001, Mann-Whitney *U* test; 3–16 sections/mouse, *n* = 4 mice/group). G) C57BL/6 mice were treated daily with RANKL (or PBS as a control) to induce M cell-differentiation and on d 2 orally-gavaged with 2x10^11^ fluorescent microbeads. Peyer’s patches and intestines were collected 24 h later to compare the functional ability of M cells in the intestines of mice from each group to acquire and transcytose particulate antigens. Fluorescent microbeads (green, arrows) were detected in sections of Peyer’s patches (left-hand panels) and intestine (right panels) by fluorescence microscopy. V, villi; broken line, lumenal surface of the gut epithelium. In the intestines of RANKL-treated mice the number of fluorescent microbeads in (H) the SED of the Peyer’s patches (*P*<0.0001, Mann-Whitney *U* test; 27 Peyer’s patch sections/mouse, n = 4 mice/group) and (I) villi (*P*<0.0001, Mann-Whitney *U* test; 24 intestine sections/mouse, n = 4 mice/group) was significantly increased. J) Sections of Peyer’s patches and villi from PBS- and RANKL-treated mice were also immunostained to identify LAMP1^+^ endosomes (red) in the epithelium. Broken lines indicate the boundary of the epithelium. K&L) Morphometric analysis of the % area of LAMP1^+^ immunostaining in (K) the FAE (1–6 FAE per mouse, *n* = 4 mice/group) and (L) villous epithelium (3–14 villi sections per mouse, *n* = 4 mice/group. DAPI (blue) was used to counterstain nuclei throughout.

Next, C57BL/6 mice (*n* = 4/group) were treated daily with RANKL to induce M cell-differentiation and tissues harvested on d 3, coincident with the peak period of induction of M cell gene expression in the gut epithelium [22, 35]. A parallel group of mice were treated with PBS as a control. IHC and morphometric analysis revealed that RANKL-treatment induced a significant increase in the number of GP2-expressing (mature) and SPIB-expressing (differentiating and mature) M cells within the FAE of Peyer’s patches (Fig 6A–6C) and also in the villous epithelium (Fig 6D–6F). This increase in M cells was associated with increased functional ability to acquire particulate antigen from the gut lumen, demonstrated by a significant increase in the number of 200 nm microbeads transcytosed into the SED of Peyer’s patches and villous cores 24 h after their administration by oral gavage (Fig 6G–6I). Although a small increase in the area of LAMP1^+^ immunostaining was observed in the FAE after RANKL treatment, the abundance of LAMP1^+^ immunostaining was unchanged in the villous epithelium (Fig 6J–6L).

We also determined whether RANKL-treatment affected other important parameters considered to be required for prion infection. IHC and morphometric analysis suggested there was no significant difference in the area of CD21/35^+^ (indicative of FDC size) or PrP^C+^ immunostaining in the Peyer’s patches (S2A–S2C Fig) or MLN (S2D & S2E Fig) of RANKL-treated mice when compared to PBS-treated controls. This implied that RANKL-treatment had no significant effect on FDC status in the Peyer’s patches or MLN.

**Fig 7:**
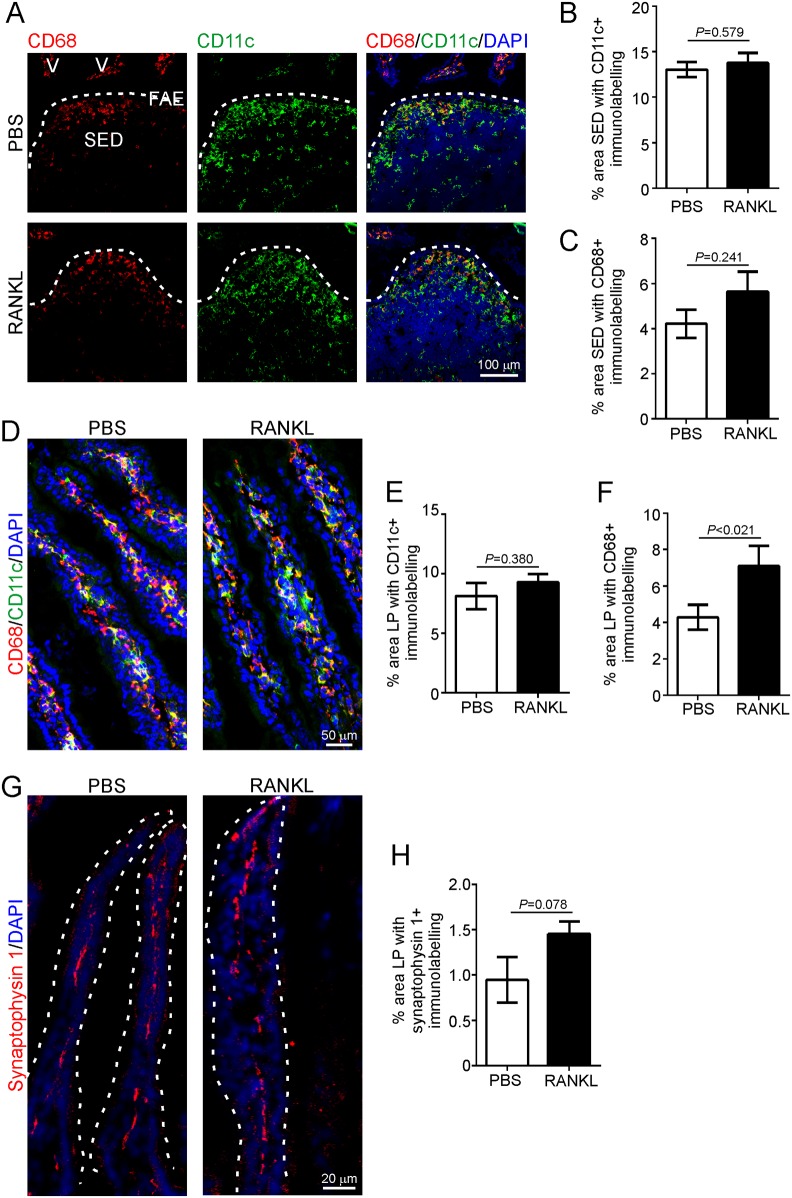
Effect of RANKL-treatment on mononuclear phagocytes and innervation in Peyer’s patches and the lamina propria of the intestine. C57BL/6 mice were treated daily with RANKL (or PBS as a control) to induce M cell-differentiation and Peyer’s patches and intestines collected on d 3. A) Immunohistochemical (IHC) comparison of the distribution of CD11c^+^ (green) and CD68^+^ (red) mononuclear phagocytes (indicative of classical DC and tissue macrophages, respectively) in Peyer’s patches of RANKL- and PBS-treated mice. Sections were counterstained with DAPI (blue) to detect cell nuclei. Broken lines show the lumenal boundary of the follicle associated epithelium (FAE). SED, subepithelial dome. Morphometric analysis suggested that the % area of the SED occupied by CD11c^+^ (B) and CD68^+^ (C) immunostaining was similar in Peyer’s patches from each group (CD11c, *P* = 0.579, Student’s *t*-test; CD68, *P* = 0.241, Mann-Whitney *U* test; 4–8 SED/mouse, *n* = 4 mice/group). D) IHC comparison of the distribution of CD11c^+^ (green) and CD68^+^ (red) mononuclear phagocytes in the lamina propria (LP) of RANK^F/F^ and RANK^ΔIEC^ mice. Sections were counterstained with DAPI (blue). Morphometric analysis suggested that the % area of (E) CD11c^+^ immunostaining was similar in the LP of mice from each group (*P* = 0.380, Student’s *t*-test), whereas the % area of (F) CD68^+^ immunostaining was increased in the LP of RANKL-treated mice (*P* < 0.021. Mann-Whitney *U* test; data derived from 4 LP areas/mouse, *n* = 4 mice/group). G) Sections of intestines from RANKL- and PBS-treated mice were immunostained to identify synaptophysin 1 (red) to enable enteric innervation in the gut wall to be compared in each treatment group. Sections were counterstained with DAPI (blue). H) Morphometric analysis suggested that the % area of synaptophysin 1^+^ immunostaining within the LP was similar (*P* = 0.078, Student’s *t*-test; data derived from 2–4 LP areas/mouse, *n* = 4 mice/group).

IHC and morphometric analysis also indicated that the % area of CD11c^+^ immunostaining in the SED of the Peyer’s patches (Fig 7A & 7B) and the LP (Fig 7D & 7E) did not differ between tissues from PBS- and RANKL-treated mice. Although the % area of CD68^+^ immunostaining was equivalent in the SED of the Peyer’s patches (Fig 7A & 7C), a significant increase was observed in the LP of RANKL-treated mice (Fig 7D & 7F). No difference in the % area of synaptophsyin 1^+^ immunostaining was observed in the LP (Fig 7G & 7H), suggesting that RANKL-treatment did not significantly affect the magnitude of the enteric innervation in the intestine.

Together, these data demonstrate that RANKL-treatment promotes M cell-differentiation in the FAE of Peyer’s patches and villous epithelium without significant effects on other key cells (FDC, CD11c^+^ cells and enteric nerves) considered to play an important role in oral prion disease pathogenesis.

### Increased M cell-density enhances susceptibility to oral prion disease

**Fig 8:**
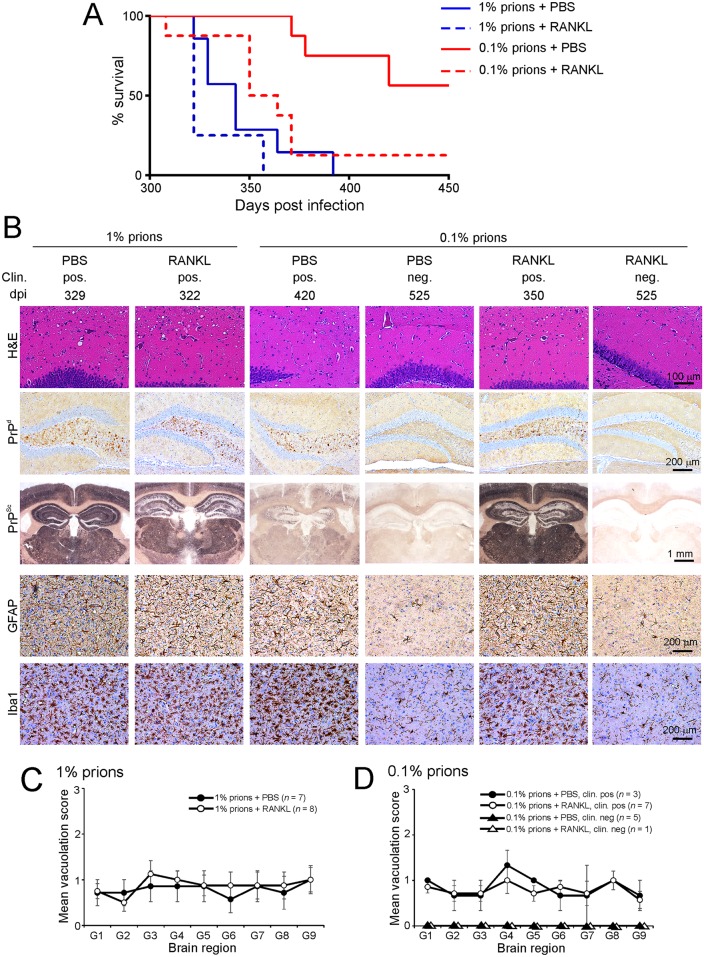
RANKL treatment significantly enhances susceptibility to oral prion disease. C57BL/6 mice were treated daily for 4 d with RANKL (or PBS as a control) to induce M cell-differentiation, and orally-exposed to a moderate (1% scrapie brain homogenate) or limiting (0.1%) dose of ME7 scrapie prions between the 3^rd^ and 4^th^ treatments. A) RANKL-treatment significantly increased disease susceptibility following oral exposure to a limiting dose of prions. Clinical prion disease survival curves for PBS- (solid lines) or RANKL-treated mice (broken lines) orally exposed to either a 1% (blue lines) or 0.1% (red lines) dose of prions (PBS/1% vs. RANKL/1%, *P* = 0.120; PBS/0.1% vs. RANKL/0.1%, *P*<0.0078; PBS/1% vs. RANKL/0.1%, *P* = 0.205; Log-rank [Mantel-Cox] test). B) High levels of spongiform pathology (H&E, upper row), heavy accumulations of PrP^d^ (brown, second row) and disease-specific PrP^Sc^ (PET immunoblot, black, third row), reactive astrocytes expressing GFAP (brown, fourth row) and active microglia expressing Iba1 (brown, bottom row) were detected in the brains of all orally-exposed mice with clinical prion disease. However, none of these histopathological signs of prion disease were detected in the brains of any of the clinically-negative mice up to at least 525 d after oral exposure. Clin., clinical prion disease status; pos., clinically positive; neg., clinically negative; individual survival times are shown (dpi, days post infection). Sections were counterstained with haematoxylin to detect cell nuclei (blue). C&D) The severity and distribution of the spongiform pathology (vacuolation) within each brain was scored on a scale of 1–5 in nine grey matter areas: G1, dorsal medulla; G2, cerebellar cortex; G3, superior colliculus; G4, hypothalamus; G5, thalamus; G6, hippocampus; G7, septum; G8, retrosplenial and adjacent motor cortex; G9, cingulate and adjacent motor cortex; Each point represents the mean vacuolation score ± SD.

To determine whether increased M cell-density in the intestine altered oral prion disease susceptibility, groups of C57BL/6 mice were treated daily with RANKL (or PBS as a control) for 4 d as above, and between the 3^rd^ and 4^th^ treatments (coincident with the peak period of induction of M-cell gene expression in the gut epithelium [22, 35]) the mice were orally exposed to either a moderate (1%) or limiting (0.1%) dose of ME7 scrapie prions. Exposure of C57BL/6 mice to a 1% dose of prions typically yields a clinical disease incidence of 100% in the recipients, whereas a 0.1% dose has a much lower incidence allowing the effects of RANKL-treatment on both survival time and prion disease susceptibility to be assessed. As anticipated, following oral exposure to a moderate (1%) dose of ME7 scrapie prions, all PBS and RANKL-treated mice developed clinical disease. However, the RANKL-treated mice succumbed to clinical disease approximately 17 d earlier with a shorter mean survival time when compared to PBS-treated control mice (PBS-treated mice, mean 346±25 d, median 343 d, *n* = 7/7; RANKL-treated mice, mean 329±18 d, median 322 d, *n* = 8/8; Fig 8A). When mice were orally exposed to a limiting (0.1%) dose of prions only three of eight PBS-treated mice succumbed to clinical disease with individual survival times of 371, 378 and 420 d (Fig 8A). The five remaining PBS-treated mice did not develop clinical prion disease up to 525 dpi. In contrast, RANKL-treatment significantly enhanced prion disease pathogenesis as seven of eight RANKL-treated mice exposed to a limiting dose of prions succumbed to clinical disease with significantly shorter survival times (Fig 8A; RANKL-treated mice, mean 352±22 d, median 350 d, *n* = 7/8; *P*<0.0078, Log-rank [Mantel-Cox] test). Only one of the eight RANKL-treated mice exposed to a limiting dose of prions was free of the clinical signs of prion disease up to at least 525 dpi.

The brains of all mice that developed clinical signs of prion disease in each treatment group had the characteristic spongiform pathology (vacuolation), astrogliosis, microgliosis and PrP^Sc^ accumulation typically associated with terminal infection with ME7 scrapie prions (Fig 8B). The distribution and severity of the spongiform pathology was also similar in the brains of all the clinically-affected mice (Fig 8C & 8D), indicating that RANKL treatment did not alter the course of CNS prions disease after neuroinvasion had occurred. In contrast, no histopathological signs of prion disease were detected in the brains of any of the clinically-negative mice.

As expected, at the terminal stage of disease high levels of PrP^Sc^ were maintained upon FDC in the Peyer’s patches, MLN and spleen of all clinically-affected mice. However, no evidence of PrP^Sc^ accumulation within these lymphoid tissues was observed in any of the orally-exposed clinically-negative mice (S3 Fig). These data show that all the clinically-negative mice were free of prions in their lymphoid tissues and brains, and therefore highly unlikely to succumb clinical prion disease after substantially extended survival times, had the observation period been extended beyond 525 dpi.

Our data suggested that RANKL-treatment significantly increased susceptibility to orally-administered prions. Indeed, no significant difference in disease incidence or mean survival time was observed in the RANKL-treated mice exposed to a 0.1% dose of prions when compared to PBS-treated control mice given a 10X higher (1%) dose (PBS/1% vs. RANKL/0.1%, *P* = 0.205; Log-rank [Mantel-Cox] test; Fig 8A). Together, these data demonstrate that increased M cell-deficiency in the gut epithelium following RANKL-treatment significantly enhances oral prion disease susceptibility by approximately 10-fold. Although certain concurrent pathogen infections or inflammatory stimuli may have multiple effects on the gut epithelium, our data suggest that factors such as these that modify M cell-density in the intestine [25, 39, 40] may represent important risk factors which can significantly influence susceptibility to orally-acquired prion infections.

### Increased M cell-density enhances the early accumulation of prions in lymphoid tissues

**Fig 9:**
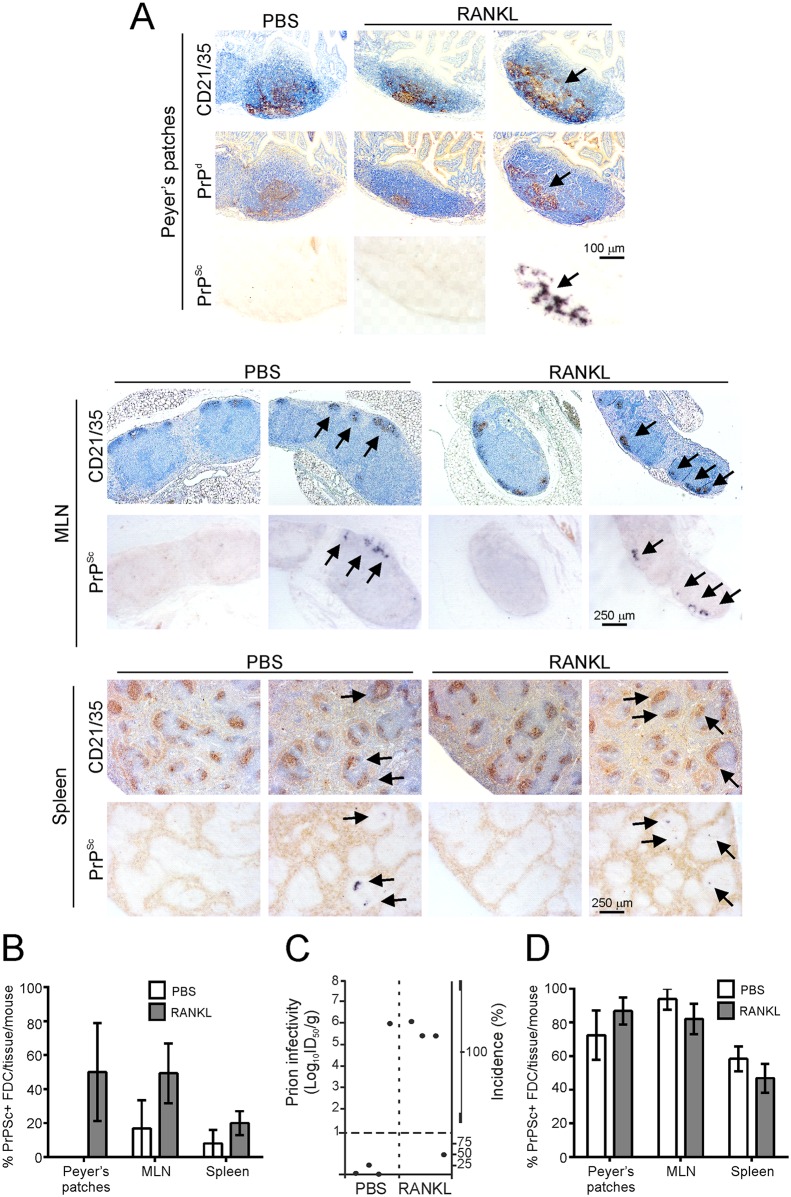
RANKL-treatment induces the earlier accumulation of prions in lymphoid tissues after oral exposure. C57BL/6 mice were treated daily for 4 d with RANKL (or PBS as a control) to induce M cell-differentiation, and orally-exposed to a moderate (1%) dose of ME7 scrapie prions between the 3^rd^ and 4^th^ treatments. Peyer’s patches, mesenteric lymph nodes (MLN) and spleens were collected at 70 and 105 days post infection (dpi). A) At 70 dpi, abundant accumulations of PrP^Sc^ (PET immunoblot, black, arrows) were clearly evident in association with FDC (CD21/35^+^ cells, brown, arrows) in the Peyer’s patches, MLN and spleens of many of the RANKL-treated mice, but were undetectable in the majority of the tissues from the PBS-treated mice. Sections were counterstained with haematoxylin to detect cell nuclei (blue). B) At 70 dpi, the frequency of FDC networks containing PrP^Sc^ was increased in the Peyer’s patches, MLN and spleens of the RANKL-treated mice when compared to PBS-treated control mice (*n* = 4 mice/group). C) Prion infectivity levels were assayed in spleens from RANKL-treated and PBS-treated control mice (*n* = 4 spleens/group) collected at 70 dpi. Prion infectivity titres (log_10_ ID_50_/g tissue) were determined by injection of tissue homogenates into groups of tga20 indicator mice (*n* = 4 recipient mice/spleen). Each symbol represents data derived from an individual spleen. Data below the horizontal line indicate disease incidence in the recipient mice <100% and considered to contain trace levels of prion infectivity. D) At 105 dpi, no difference in the frequency of FDC networks containing PrP^Sc^ was observed in the Peyer’s patches, MLN and spleens of mice from each treatment group (*n* = 4 mice/group).

Prion replication within Peyer’s patches is essential for efficient neuroinvasion after oral exposure [10–13]. We therefore determined whether the decreased survival times and increased prion disease susceptibility in orally-exposed RANKL-treated mice were associated with the earlier accumulation of prions in their lymphoid tissues. Mice were treated with RANKL (or PBS as a control) and orally exposed to a 1% dose of ME7 scrapie prions as above, and culled at intervals afterwards (*n* = 4/group). Abundant accumulations of PrP^Sc^ were clearly evident in association with FDC in the Peyer’s patches, MLN and spleen of RANKL-treated mice by 70 dpi, and were undetectable in the majority of the tissues from the PBS-treated animals at this time (Fig 9A & 9B). To compare prion infectivity levels between the treatment groups, spleen homogenates were prepared and injected intracerebrally (i.c.) into groups of tga20 indicator mice (*n* = 4/spleen homogenate). As the expression level of PrP^C^ controls the prion disease incubation period, tga20 mice which overexpress PrP^C^ are extremely useful as indicator mice in prion infectivity bioassays as they succumb to disease with much shorter survival times than conventional mice [59]. Significantly high levels of prion infectivity were detected in three of four of the spleens collected from the RANKL-treated mice at 70 dpi, whereas only one of four spleens from the PBS treated spleen contained detectable levels of prion infectivity (*P*<0.0002, Log-rank [Mantel-Cox] test; Fig 9C). By 105 dpi abundant accumulations of PrP^Sc^ were detected at equivalent frequencies in the lymphoid tissues of the PBS- and RANKL-treated animals (Fig 9D).

These data show that an increased density of M cells in the intestinal epithelium at the time of oral exposure enhanced the uptake of prions from the gut lumen, as the RANKL-treated mice accumulated prions within their lymphoid tissues significantly earlier than control mice.

### The effects of RANKL treatment on oral prion disease pathogenesis are restricted to the intestinal epithelium

Although a rare occurrence in the steady-state, certain pathogenic microorganisms can stimulate the direct sampling of the gut lumenal contents by classical DC [60–63]. Whether this direct sampling activity by classical DC also contributes to the efficient uptake of orally-administered prions in the steady-state is uncertain [8, 16, 49]. Since RANKL was administered systemically in the current study, it is plausible that this treatment may have stimulated the direct sampling of the lumenal contents by cells other than M cells such as classical DC. An additional experiment was performed to test this hypothesis. As shown above, RANK^ΔIEC^ mice are unable to accumulate prions in their Peyer’s patches due to the specific lack of M cells (Fig 5). Since RANK-deficiency in RANK^ΔIEC^ mice is restricted only to intestinal epithelial cells [23], we reasoned that if the effects of RANKL-treatment on disease pathogenesis were independent of their effects on M cells, then RANKL-treatment would also facilitate the uptake of prions into the Peyer’s patches of RANK^ΔIEC^ mice. To address this issue, RANK^ΔIEC^ mice were treated with RANKL and orally exposed to a 1% dose of ME7 scrapie prions as in the previous experiment. At 105 dpi Peyer’s patches and MLN were collected and analysed for the presence of PrP^Sc^ as before. As anticipated, abundant accumulations of PrP^Sc^ were detected in association with FDC in the Peyer’s patches and MLN of orally-exposed C57BL/6 wild-type (WT) control mice by 105 dpi. However, no PrP^Sc^ was detected in tissues from RANKL-treated RANK^ΔIEC^ mice (S4 Fig). These data clearly show that RANKL-treatment was unable to restore prion accumulation in the Peyer’s patches and MLN of RANK^ΔIEC^ mice, indicating that the major effects of RANKL-treatment on oral prion disease pathogenesis were due to effects on M cell-deficiency in the intestinal epithelium.

### Increased M cell-density in the FAE of Peyer’s patches, not the villous epithelium, is responsible for the increased oral prion disease susceptibility in RANKL-treated mice

RANKL-treatment stimulates M cell-differentiation within the FAE of the Peyer’s patches and also in the villous epithelium (Fig 6; [22, 35, 64]). We therefore considered it plausible that the enhanced prion pathogenesis we observed in RANKL-treated mice was due to the increased uptake of prions by the M cells induced in the villous epithelium. If RANKL-treatment had stimulated the uptake of prions predominantly via villous M cells, we reasoned that this would have facilitated the earlier transport of prions directly to the MLN [65]. An additional experiment was designed to test this hypothesis.

**Fig 10:**
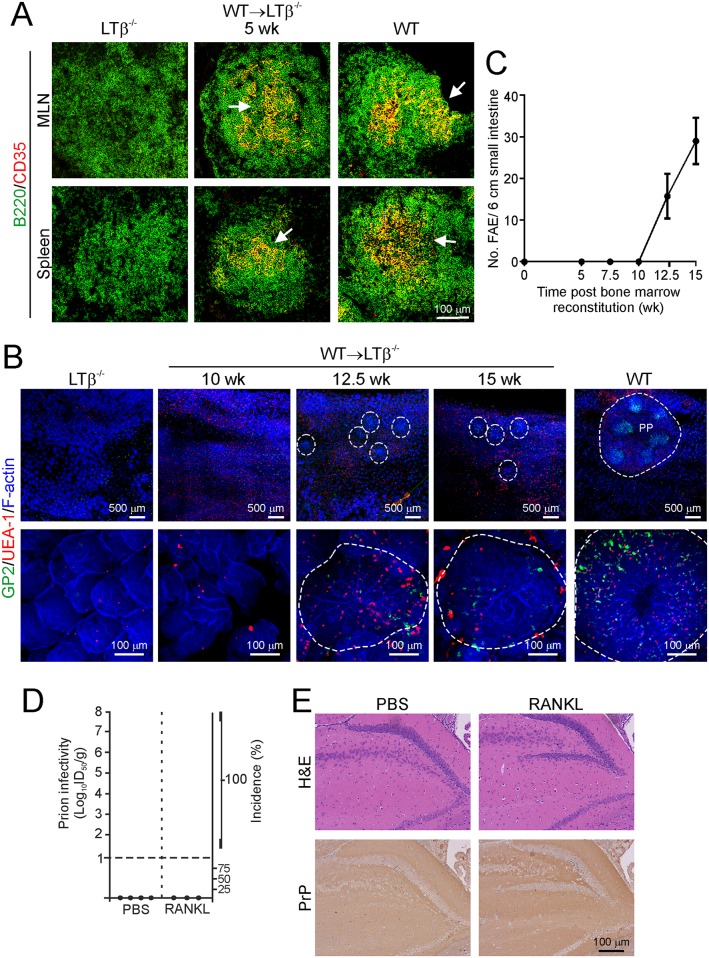
Villous M cells induced by RANKL-treatment do not enhance the transport prions to the mesenteric lymph nodes (MLN). This experiment aimed to determine whether the enhanced prion pathogenesis we observed in RANKL-treated C57BL/6 mice wild-type (WT) mice was due to the increased uptake of prions by the M cells induced in the villous epithelium. A mouse model was created in which their small intestines lacked follicle associated epithelia (FAE) and M cell-containing gut-associated lymphoid tissues, but possessed follicular dendritic cells (FDC) in their MLN. A-C) Peyer’s patches-deficient LTβ^-/-^ mice were γ-irradiated and reconstituted with WT bone marrow (WT→LTβ^-/-^ mice) and tissues from 3–4 mice examined at intervals afterwards. LTβ^-/-^ mice and WT mice were included as controls. A) Immunohistochemical analysis revealed that the development of FDC (CD35^+^ cells, red) in the B cell-follicles (B220^+^ cells, green) of MLN (upper panels) and spleens of WT→LTβ^-/-^ mice was restored by 5 wk after bone-marrow reconstitution. Images are representative of 3–4 mice/group. B) Beginning at 5 wk post-reconstitution, and at 2.5 wk intervals thereafter, 3 individual 2 cm pieces of intestine/mouse were whole-mount immunostained to detect M cells (GP2^+^ cells, green) and goblet cells (UEA-1^+^ cells, red). F-actin (blue) was used as a counterstain. Characteristic epithelial structures that contained GP2^+^ M cells and resembled the FAE covering isolated lymphoid follicles were absent in the intestines of LTβ^-/-^ mice (left-hand panels) but were detectable from 12.5 wk post-reconstitution in WT→LTβ^-/-^ mice. FAE highlighted by broken lines in WT→LTβ^-/-^ panels. The broken line in the upper right-hand WT panel highlights 6 individual FAE covering a Peyer’s patch. Images are representative of 3–4 mice/group. C) The total no. FAE across the 3 individual 2 cm intestinal pieces were counted (*n* = 3–4 mice/group). D) WT→LTβ^-/-^ mice were prepared as above and at 7.5 wk post reconstitution (when FDC present but FAE absent) were treated with RANKL for 4 d (or PBS as a control) to induce the differentiation of villous M cells in the intestine. Between the 3^rd^ and 4^th^ treatments, the mice were subsequently orally-exposed to a 1% dose of ME7 scrapie prions, and MLN were collected 28 d later (*n* = 3–4 MLN/group). Prion infectivity titres (log_10_ ID_50_/g tissue) were determined by injection of MLN tissue homogenates into groups of 4 tga20 indicator mice. Each symbol represents data derived from an individual MLN. Data below the horizontal line indicate disease incidence in the recipient mice <100% and considered to contain trace levels of prion infectivity. E) Confirmation that none of the brains from the clinically-negative tga20 indicator mice injected with MLN from PBS- or RANKL-treated donors contained histopathological signs of prion disease at the end of the experiment (200 d post-injection): spongiform pathology (H&E, upper panels), or PrP^d^ deposition (lower panels). Sections were counterstained with haematoxylin to detect cell nuclei (blue). Images are representative of 12–16 mice/group.

Lymphotoxin-β-deficient (LTβ^-/-^) mice lack Peyer’s patches and most peripheral lymph nodes, but retain MLN and the spleen [66]. These mice also lack FDC in their remaining lymphoid tissues, as constitutive LT-stimulation is essential for their maintenance [67], and are refractory to oral prion infection [10, 11]. Peyer’s patches-deficient LTβ^-/-^ mice were γ-irradiated and reconstituted with LTβ-expressing (WT) bone marrow (termed WT→LTβ^-/-^ mice, hereinafter) and tissues collected at 2.5 weekly intervals (*n* = 4 mice/group). Although the formation of FDC networks within the MLN and spleens of WT→LTβ^-/-^ mice is restored by 5 wk after reconstitution (Fig 10A), WT→LTβ^-/-^ mice remain refractory to oral prion disease [11] as Peyer’s patches, not the MLN, are the essential early sites of prion accumulation and neuroinvasion after oral exposure [11, 13].

The reconstitution of LTβ^-/-^ mice with WT bone marrow also induces the differentiation and maturation of isolated lymphoid follicles (ILF) throughout the small intestine [11, 68, 69]. Mature ILF characteristically contain a single organized B cell-follicle, a network of FDC, and an M cell-containing FAE at the lumenal surface [11, 13, 68]. Since we have shown that mature small intestinal ILF are important sites of prion accumulation and neuroinvasion [11, 13], it was necessary to ensure there were no ILF with M cell-containing FAE in the intestines of WT→LTβ^-/-^ mice at the time of RANKL-treatment and prion exposure. Whole-mount immunostaining of three individual 2 cm sections of small intestine from each WT→ LTβ^-/-^ mouse showed that ILF with developed FAE containing GP2^+^ M cells were not present until 12.5 post-reconstitution (Fig 10B & 10C). These data revealed a window of opportunity between 5–10 wk post-reconstitution during which the small intestines of WT→LTβ^-/-^ mice lacked FAE and M cell-containing GALT, but possessed FDC within their MLN. This FAE-deficient model was therefore used to determine whether RANKL-treatment facilitated the direct delivery of prions from the gut lumen to the MLN.

At 7.5 wk post-reconstitution WT→LTβ^-/-^ mice (*n* = 3-4/group) were treated with RANKL (or PBS as a control) for 4 d and orally-exposed to prions as before, and prion infectivity levels determined in their MLN 28 d later. Tissues were assayed for prion infectivity at this time after oral exposure to determine whether RANKL-treatment of WT→LTβ^-/-^ mice facilitated the earlier replication of prions within the MLN. Consistent with our previous data showing that Peyer’s patches in the small intestine, not the MLN, are the important early sites of prion accumulation after oral exposure [11, 13], prion infectivity was undetectable in the MLN of the PBS control-treated WT→LTβ^-/-^ mice. Similarly, prion infectivity was also undetectable in the MLN of the RANKL-treated WT→LTβ^-/-^ mice. In each instance all the recipient tga20 indicator mice (*n* = 4/MLN homogenate tested) were free of clinical disease up to 200 dpi (Fig 10D) and had no histopathological signs of prion disease in their brains (spongiform pathology and PrP^d^ deposition; Fig 10E). These data clearly show that RANKL-treatment did not stimulate the early transport of prions directly to the MLN. This suggests that the enhanced prion disease pathogenesis observed in RANKL-treated mice was due to the increased uptake of prions from the gut lumen by M cells in the FAE of the Peyer’s patches, rather than by villous M cells.

## Discussion

Here we show that the density of M cells in the gut epithelium directly influences oral prion disease pathogenesis and susceptibility. In the specific absence of M cells, the accumulation of prions in Peyer’s patches and subsequent neuroinvasion was blocked, demonstrating that prion translocation across the gut epithelium in association with M cells is essential to establish host infection. Our data also imply that an absence or reduction in M cell-abundance may significantly reduce susceptibility to many naturally acquired prion diseases such as vCJD in humans, CWD in cervids and natural sheep scrapie. For example, in the UK most clinical vCJD cases have predominantly occurred in young adults (median age at death, ~28 years) [4], but epidemiological data indicate that this age-related susceptibility is not simply due to the exposure of young individuals to greater levels of the BSE agent through dietary preference [70]. We have previously shown that the density of functionally mature M cells in the Peyer’s patches of aged mice is substantially reduced [71], suggesting that the reduced susceptibility of aged mice to oral prion infection [72] is at least in part due to the inefficient uptake of prions from the gut lumen by M cells.

We also show that increased M-cell density at the time of oral exposure exacerbated prion disease pathogenesis: the uptake of prions from the gut lumen was enhanced, and as a consequence, survival times were decreased and disease susceptibility was increased approximately 10-fold. The density of M cells in the gut epithelium can be modified by the presence of certain pathogenic bacteria or inflammatory stimuli [25, 39, 40]. Although these stimuli may have multiple effects on the gut epithelium which can influence the integrity of this barrier, data in the current study provide a significant advance in our understanding of how factors that increase the density of M cells in the gut epithelium may increase susceptibility to orally-acquired prion infection. For example, the enteroinvasive bacterium *Salmonella* Typhimurium can specifically and rapidly transform enterocytes in the FAE of Peyer’s patches into M cells in order to facilitate host infection [25]. Furthermore, an independent study has shown that concurrent infection with *S*. Typhimurium significantly increased oral prion disease susceptibility [43]. Although this observation was originally attributed to the colitis induced by the pathogen in the large intestine, data in the current study suggest a role for effects on M cells in the small intestine cannot be excluded.

During the BSE epidemic in the UK it is estimated that approximately 500,000 infected cattle were slaughtered for human consumption [73]. Despite the widespread dietary exposure of the UK human population to BSE prions, clinical cases of vCJD have fortunately been rare (Ref. [4]; 178 definite or probable cases, as of 5^th^ December 2016; www.cjd.ed.ac.uk/documents/figs.pdf). This implies that the ability to acquire prions from the gut lumen may differ between individuals. Studies using transgenic mice expressing human PrP^C^ suggest that the transmission of BSE to humans is restricted by a significant species barrier [74]. After interspecies prion exposure, the processing and amplification of prions upon FDC in lymphoid tissues is important for their adaptation to the new host and to achieve neuroinvasion [75, 76]. Thus, it is plausible that factors which increase the density of M cells in the small intestine may enable a greater burden of prions to enter Peyer’s patches, increasing the probability that more will be able to avoid clearance by cells such as macrophages, [11, 77]. This may provide a greater opportunity for prion quasi-species present within the original inoculum with zoonotic potential to be selected and undergo adaptation and amplification upon FDC [78]. These effects may help to reduce the transmission barrier to some orally acquired prion strains.

Enterocytes within the FAE overlying the Peyer’s patches specifically contain large LAMP1^+^ endosomes [16]. A detailed high resolution IHC-based study has shown that within the first day following oral exposure of mice to prions, PrP^Sc^ was detected within these large LAMP1^+^ endosomes of FAE enterocytes, and to a lesser extent in M cells [16]. These FAE enterocyte-associated endosomes have been proposed as a potential M cell-independent route through which lumenal proteins and prions may also be taken up into Peyer’s patches [16]. In the current study the presence and abundance of the large LAMP1^+^ endosomes within FAE enterocytes was unaffected in M cell-deficient RANK^ΔIEC^ mice. These data clearly show that the specific lack of M cells in the FAE, rather than an absence of the large LAMP1^+^ endosomes within FAE enterocytes, was responsible for the blocked prion accumulation in Peyer’s patches. Furthermore, the accumulation of prions in the Peyer’s patches, MLN and spleens of orally-exposed M cell-deficient RANK^ΔIEC^ mice was undetectable up to at least 440 d after exposure. As abundant prion accumulation is typically evident in these tissues in conventional (WT) mice by 105 d after exposure, this implies that in the absence of M cells, any prions that do enter the Peyer’s patches via alternative routes may be of insufficient magnitude to establish infection. Indeed PrP^Sc^ was also undetectable in the lymphoid tissues and CNS of these mice up to at least 440 dpi. Instead the prions that are acquired from the gut lumen by these M cell-independent routes are most likely sequestered and destroyed by cells such as macrophages, which are considered to degrade prions [77], rather than being efficiently transported to FDC where they undergo amplification before neuroinvasion [7, 10, 13, 15]. RANK^ΔIEC^ mice show reduced IgA production and delayed germinal centre responses in their Peyer’s patches [23]. This suggests that antigens that are transcytosed by M cells are preferentially targeted to the FDC-containing B-cell follicles to initiate antibody responses. Therefore, M cells, in contrast to FAE enterocytes with large LAMP1^+^ endosomes, may be considered to facilitate the efficient transfer of prions from the gut lumen to FDC in the B-cell follicles of Peyer’s patches.

A separate IHC-based study also has proposed that the uptake of scrapie-affected brain homogenate across the jejunal epithelium of lambs occurs independently of M cells [34]. However, if prions do efficiently establish infection within Peyer’s patches after their translocation across the gut epithelium by enterocytes, one would not expect the specific absence of M cells in RANK^ΔIEC^ mice to block oral prion disease susceptibility. In the above *in vivo* study [34], large quantities of scrapie-affected brain homogenate were injected directly into the lumen of ligated loops of jejunum. The presence of a large bolus of prions concentrated within the lumen of these ligated loops may have facilitated prion uptake into alternative cellular compartments to those utilized following exposure to physiologically relevant doses via the oral cavity. Although evidence of prions (PrP^d^) was detected in the underlying LP of these lambs, it was interesting to note that no intraepithelial PrP^d^ accumulations were detected by IHC [34]. Whether the prions were transiently present in enterocytes and/or M cells soon after exposure, but at levels below the reliable detection limit or in a conformation which could not be detected by the IHC protocols used, remains to be determined. By comparison, in the study by Kujala and colleagues discussed above [16], PrP^Sc^ was detected within the FAE during the first day after oral exposure using highly sensitive cryo-immunogold electron microscopy. M cells unlike the neighbouring enterocytes have a very narrow cytoplasm due to the presence of the MNP-containing basolateral pocket [20]. Thus it is also plausible that the prion transit time through M cells may be extremely rapid, restricting the ability of IHC to reliably detect low levels of prions or other particles which are being transcytosed through them. Surgical manipulation and manual compression of the intestine can temporarily inhibit intestinal motility and induce intestinal inflammation with activation of resident macrophages, as occurs during postoperative ileus [79, 80]. These factors may have a significant influence on the uptake of prions from the lumen of surgically-ligated intestinal loops.

Using extremely sensitive PrP^Sc^-based detection assays, two independent studies reported the presence of low/trace levels of prions in the blood-stream within minutes of oral exposure [81, 82]. The cellular route through which the prions initially gained access to the blood-stream was not determined in these studies. Urayama and colleagues [82] suggested that the levels of PrP^Sc^ that initially contaminated the blood-stream after oral exposure were sufficient to initiate infection in the brain. However, data from several studies show that prion replication upon FDC in Peyer’s patches in the small intestine is essential to establish host infection after oral exposure [7, 8, 10–13]. Furthermore, in the temporary absence of FDC at the time of oral exposure, prion disease susceptibility is blocked [6]. Thus although PrP^Sc^ may be detected in the blood-stream soon after oral exposure using highly sensitive assays [81, 82], data elsewhere indicate that the levels of prions that are initially within it are unable to directly establish host infection and achieve neuroinvasion.

After uptake by M cells, CD11c^+^ classical DC are considered to deliver prions towards FDC, as their transient depletion reduces susceptibly to oral prion disease [8]. A partial reduction in CD11c^+^ immunostaining was observed in the SED of Peyer’s patches from RANK^ΔIEC^ mice, implying a partial reduction in the abundance of these cells. M cells specifically express the chemokine CCL9 [22] which mediates the attraction of certain classical DC populations towards the FAE [83]. Thus, the reduced CD11c^+^ immunostaining in the SED of RANK^ΔIEC^ mice may be a consequence of the absence of attraction of CD11c^+^ cells towards the basolateral pockets of M cells. This partial reduction in CD11c^+^ immunostaining in SED region alone could not account for the complete block of prion accumulation observed in RANK^ΔIEC^ mice, as our previous data show that the depletion of CD11c^+^ cells (>85%) prior to oral exposure does not block neuroinvasion [8]. Although the germinal centre response is delayed in RANK^ΔIEC^ mice [23], our data suggested that FDC status was unaffected in these mice. Furthermore, the FDC in the Peyer’s patches, MLN and spleen of these mice were capable of accumulating high levels of PrP^Sc^ after injection of prions by the i.p. route. We have also previously shown that an absence of germinal centres themselves does not influence peripheral prion disease pathogenesis [84].

The GALT in the small intestine such as the Peyer’s patches, not those in the large intestine, are the major early sites of prion uptake, replication and neuroinvasion after oral exposure [11, 13, 16]. RANKL-RANK signalling is also necessary for the induction of M cell-differentiation within the large intestine, but in contrast to its role in the small intestine, it does not induce their maturation. As a consequence, GP2-expressing functionally mature M cells are scarce in the FAE overlying the large intestinal GALT [64]. Consistent with this, systemic RANKL-treatment also does not increase the abundance of functionally mature M cells in the FAE overlying the caecal patches or in the conventional epithelium of large intestine [64]. These data suggest that the effects of systemic RANKL-treatment on oral prion disease pathogenesis observed in the current study were due to an increased abundance of mature M cells specifically in the small intestine.

In the steady state, functionally mature M cells are confined to the FAE overlying the Peyer’s patches and are extremely rare within the villous epithelium. However, systemic RANKL-treatment, as used here, significantly increases the abundance of mature M cells in the FAE overlying Peyer’s patches and throughout the villous epithelium [22, 35, 64]. Therefore, it is plausible that the effects of systemic RANKL-treatment on oral prion disease pathogenesis were in part due to the enhanced uptake of prions by villous M cells, facilitating their more efficient delivery to the MLN. Using LTβ^-/-^ mice reconstituted with WT bone marrow (WT→LTβ^-/-^ mice), we generated mice that lacked Peyer’s patches or other M cell-containing GALT structures (ILF) in their small intestines, but retained MLN which contained mature FDC. If the major effect of RANKL-treatment on oral prion pathogenesis was due to uptake by villous M cells and enhanced delivery from the LP to the MLN, the accumulation of prions in the MLN would likewise be enhanced in these mice after RANKL-treatment. However, our data clearly show that RANKL-treatment did not enhance the accumulation of prions within the MLN of WT→LTβ^-/-^ mice. This demonstrates that the major effect of RANKL-treatment on oral prion disease pathogenesis and susceptibility was due to the increased uptake of prions across the FAE overlying the Peyer’s patches in the small intestine. The absence of detectable levels of prion infectivity in the MLN at the time of analysis suggests that any low levels of prions that do reach this tissue immediately after oral exposure are either not delivered to FDC in the MLN as efficiently as they are in the Peyer’s patches, or are of insufficient magnitude to establish infection on FDC and are thus most likely degraded by macrophages [11, 77]. Our IHC analysis implied that the abundance of CD68^+^ macrophages was increased in the LP after RANKL-treatment, suggesting that it is also plausible that any prions that had been acquired by villous M cells were subsequently sequestered and destroyed in the LP by macrophages. Classical DC in the LP of the intestine are considered to deliver lumenal antigens directly to MLN [65]. Here, RANKL-treatment of RANK^ΔIEC^ mice did not restore prion accumulation in their Peyer’s patches and MLN following oral exposure, demonstrating that RANKL-treatment did not alter the uptake of prions from the gut lumen by non-epithelial cells, such as classical DC. Our data suggest that direct sampling of the lumenal contents by classical DC in the LP [60–63] is also unlikely to contribute significantly to prion uptake from the gut lumen, as this too would result in the direct delivery of prions to the MLN [65].

In conclusion, we show that the initial uptake and transfer of prions across the gut epithelium in association with M cells is essential to establish host infection. Importantly, we also demonstrate that the density of M cells in the FAE overlying the Peyer’s patches in the small intestine directly controls the efficiency of oral prion infection. In the specific absence of M cells, the uptake and accumulation of prions in Peyer’s patches and their subsequent spread to the MLN and spleen is blocked. In contrast, oral prion disease susceptibility was enhanced approximately 10-fold in mice in which M cell-deficiency in the gut epithelium was increased. Thus, M cells could be considered as the gatekeepers of oral prion infection whose density directly limits or enhances disease susceptibility. Further studies are necessary to determine whether most orally acquired prion strains similarly exploit intestinal M cells to establish host infection after oral exposure, but data from independent *in vivo* and *in vitro* studies using mouse-passaged RML scrapie prions [30], Fukuoka-1 prions [31], BSE prions [32] and 263K hamster prions [17] imply a similar requirement. Antigen sampling M cells are also present in the FAE overlying the NALT in the nasal cavity [44, 45], but data from the analysis of prion disease pathogenesis in hamsters implies that the requirement for M cell-mediated uptake may vary depending on the route of exposure [85]. After intra-nasal exposure some transient uptake of 263K prions was observed in M cells within the FAE overlying the NALT, but a greater magnitude of paracellular transport across the epithelia within the nasal cavity was also noted [85]. Although certain concurrent pathogen infections, inflammatory stimuli and aging may have multiple effects on the gut epithelium, our data suggest that factors such as these that can modify M cell-density in the small intestine [25, 39, 40, 71] may represent important risk factors which can significantly influence susceptibility to orally-acquired prion infections. Our data also raise the possibility that the density of M cells in the gut epithelium may similarly influence susceptibility to other important orally-acquired bacterial and viral pathogens which are considered to exploit M cells to infect the host [24–28].

## Materials and Methods

### Ethics statement

All studies using experimental mice and regulatory licences were approved by both The Roslin Institute’s and University of Edinburgh’s ethics committees. All animal experiments were carried out under the authority of a UK Home Office Project Licence (PPL60/4325) within the terms and conditions of the strict regulations of the UK Home Office ‘Animals (scientific procedures) Act 1986’. Where necessary, anaesthesia appropriate for the procedure was administered, and all efforts were made to minimize harm and suffering. Mice were humanely culled by a UK Home Office Schedule One method.

### Mice

The following mouse strains were used in this study where indicated: C57BL/6J; Villin-cre (Tg(Vil-cre)997Gum/J strain; The Jackson Laboratory, Bar Harbor, ME); RANK^fl/fl^, which have *loxP* sites flanking exons 2 and 3 of *Tnfrsf11a* (which encodes RANK) [23]; LTβ^-/-^ [86]; tga20, which overexpress PrP^C^ [59]. All mice were bred and maintained on a C57BL/6J background and housed under SPF conditions.

### γ-Irradiation and bone-marrow reconstitution

Bone-marrow from the femurs and tibias of donor mice was prepared as single-cell suspensions (3x10^7^–4x10^7^ viable cells/ml) in HBSS (Life Technologies, Paisley, UK). Recipient adult LTβ^-/-^ mice (6–8 weeks old) were **γ**-irradiated (10 Gy) and 24 h later reconstituted with 100 μl bone-marrow by injection into the tail vein.

### Recombinant mouse RANKL

Glutathione *S*-transferase—RANKL fusion protein was prepared as described [35]. To enhance M-cell-density in the gut epithelium mice were treated with RANKL *in vivo* as previously described [22, 35]: d 0 injected with RANKL by a combination of i.p. and subcutaneous injection (50 μg/ea.); d 1, 50 μg RANKL by subcutaneous injection; d 2, 50 μg RANKL by subcutaneous injection; d 3, 50 μg RANKL by subcutaneous injection. Mice were orally exposed to prions or gavaged with fluorescent microbeads on d 2 after the onset of RANKL treatment.

### Prion exposure and disease monitoring

For oral exposure, mice were fed individual food pellets doused with 50 μl of a 1% (containing approximately 2.5 X 10^4^ i.c. ID_50_ units) or 0.1% (w/v) dilution of scrapie brain homogenate prepared from mice terminally-affected with ME7 scrapie prions according to our standard protocol [7–9, 11, 13, 72]. During the dosing period mice were individually housed in bedding- and food-free cages. Water was provided *ad libitum*. A single prion-dosed food pellet was then placed in the cage. The mice were returned to their original cages (with bedding and food *ad libitum*) as soon as the food pellet was observed to have been completely ingested. The use of bedding- and additional food-free cages ensured easy monitoring of consumption of the prion-contaminated food pellet. For i.p. exposure, mice were injected with 20 μl of a 1% dilution of scrapie brain homogenate. Following prion exposure, mice were coded and assessed weekly for signs of clinical disease and culled at a standard clinical endpoint. The clinical endpoint of disease was determined by rating the severity of clinical signs of prion disease exhibited by the mice. Following clinical assessment, mice were scored as “unaffected”, “possibly affected” and “definitely affected” using standard criteria which typically present in mice clinically-affected with ME7 scrapie prions. Clinical signs following infection with the ME7 scrapie agent may include: weight-loss, starry coat, hunched, jumpy behaviour (at early onset) progressing to limited movement, upright tail, wet genitals, decreased awareness, discharge from eyes/blinking eyes, ataxia of hind legs. The clinical endpoint of disease was defined in one of the following ways: i) the day on which a mouse received a second consecutive “definite” rating; ii) the day on which a mouse received a third “definite” rating within four consecutive weeks; iii) the day on which a mouse was culled in extremis. Survival times were recorded for mice that did not develop clinical signs of disease or were culled when they showed signs of intercurrent disease. Prion diagnosis was confirmed by histopathological assessment of vacuolation in the brain. For the construction of lesion profiles, vacuolar changes were scored in nine distinct grey-matter regions of the brain as described [87].

For bioassay of prion infectivity individual MLN or spleen were prepared as 1% (wt/vol) homogenates in physiological saline. For each tissue homogenate groups of tga20 indicator mice (*n* = 4/homogenate) were injected i.c. with 20 μl of each homogenate. The prion infectivity titre in each sample was determined from the mean incubation period in the indicator mice, by reference to a dose/incubation period response curve for ME7 scrapie-infected spleen tissue serially titrated in tga20 mice using the relationship: *y* = 9.4533–0.0595*x* (where *y* is log ID50 U/20 μl of homogenate, and *x* is the incubation period; *R*^2^ = 0.9562).

### IHC and immunofluorescent analyses

Whole-mount immunostaining was performed as previously described [9]. Peyer’s patches, NALT and pieces of small intestines were fixed with BD Cytofix/Cytoperm (BD Biosciences, Oxford, UK), and subsequently immunostained with rat anti-mouse GP2 mAb (MBL International, Woburn, MA; 5 μg/ml). Following addition of primary Ab, tissues were stained with Alexa Fluor 488-conjugated anti-rat IgG Ab (Life Technologies), rhodamine-conjugated *Ulex europaeus* agglutinin I (UEA-1; Vector Laboratories Inc., Burlingame, CA; 20 μg/ml) and Alexa Fluor 647-conjugated phalloidin to detect f-actin (Life Technologies; 4 U/ml).

Intestines, MLNs and spleens were also removed and snap-frozen at the temperature of liquid nitrogen. Serial frozen sections (6 μm in thickness) were cut on a cryostat and immunostained with the following antibodies: FDC were visualized by staining with mAb 7G6 to detect CR2/CR1 (CD21/35; BD Biosciences; 1 μg/ml) or mAb 8C12 to detect CR1 (CD35; BD Biosciences; 1.25 μg/ml); cellular PrP^C^ was detected using PrP-specific polyclonal antibody (pAb) 1B3 [88] (1/1000 dilution); B cells were detected using rat anti-mouse B220 mAb (clone RA3-6B2, Life Technologies; 5 μg/ml); MNP were detected using hamster anti-mouse CD11c mAb (clone N418, Bio-Rad, Kidlington, UK; 5 μg/ml) or rat anti-mouse CD68 mAb (clone FA-11, Biolegend, Cambridge, UK; 5 μg/ml); rat anti-mouse CD107a (clone 1D4B; Biolegend; 2.5 μg/ml) to detect LAMP1; nerve synapses were detected using rabbit anti-synaptophysin 1 (Synaptic Systems, Göttingen, Germany; 1/150 dilution). For the detection of SPIB in paraformaldehyde-fixed sections, antigen retrieval was performed with citrate buffer (pH 7.0, 121°C, 5 min.) prior to immunostaining with sheep anti-mouse SPIB polyclonal antibody (R&D Systems, Abingdon, UK; 0.4 μg/ml). Appropriate species and immunoglobulin isotype control Ab were used as controls (S5 Fig). Where appropriate, sections were counter-stained with DAPI (2.86 μM) to detect cell nuclei (Life Technologies).

For the detection of disease-specific PrP (PrP^d^) in intestines, MLN, spleens and brains, tissues were fixed in periodate-lysine-paraformaldehyde fixative and embedded in paraffin wax. Sections (thickness, 6 μm) were deparaffinised, and pre-treated to enhance the detection of PrP^d^ by hydrated autoclaving (15 min, 121°C, hydration) and subsequent immersion formic acid (98%) for 10 min. Sections were then immunostained with 1B3 PrP-specific pAb (1/1000 dilution). For the detection of astrocytes, brain sections were immunostained with anti-glial fibrillary acidic protein (GFAP; DAKO, Ely, UK; 1/400 dilution). For the detection of microglia, deparaffinised brain sections were first pre-treated with citrate buffer and subsequently immunostained with anti-ionized calcium-binding adaptor molecule 1 (Iba1; Wako Chemicals GmbH, Neuss, Germany; 0.5 μg/ml). For the detection of FDC in intestines, MLN and spleens, deparaffinised sections were first pre-treated with Target Retrieval Solution (DAKO) and subsequently immunostained with anti-CD21/35 mAb. PET immunoblot analysis was used to confirm the PrP^d^ detected by immunohistochemistry was proteinase K-resistant PrP^Sc^ [57]. Membranes were subsequently immunostained with 1B3 PrP-specific pAb (1/4000 dilution).

For light microscopy, following the addition of primary antibodies, biotin-conjugated species-specific secondary antibodies (Stratech, Soham, UK) were applied and immunolabelling was revealed using HRP-conjugated to the avidin-biotin complex (ABC kit, Vector Laboratories) and visualized with DAB (Sigma, Dorset, UK). Sections were counterstained with haematoxylin to distinguish cell nuclei. For fluorescent microscopy, following the addition of primary antibody, streptavidin-conjugated or species-specific secondary antibodies coupled to Alexa Fluor 488 (green), Alexa Fluor 594 (red) or Alexa Fluor 647 (blue) dyes (Life Technologies) were used. Sections were counterstained with either DAPI or Alexa Fluor 647-conjugated phalloidin and subsequently mounted in fluorescent mounting medium (DAKO). Images of whole-mount immunostained tissues and cryosections were obtained using a Zeiss LSM710 confocal microscope (Zeiss, Welwyn Garden City, UK).

### Image analysis

For morphometric analysis, images were analysed using ImageJ software (http://rsb.info.nih.gov/ij/) as described on coded sections [89]. Background intensity thresholds were first applied using an ImageJ macro which measures pixel intensity across all immunostained and non-stained areas of the images. The obtained pixel intensity threshold value was then applied in all subsequent analyses. Next, the number of pixels of each colour (black, red, green, yellow etc.) were automatically counted. For these analyses, data are presented as the proportion of positively-stained pixels for a given IHC marker per total number of pixels (all colours) in the specific area of interest (eg: SED, FAE, LP etc.). In each instance, typically 3–6 images were analysed per mouse, from tissues from multiple mice per group (*n* = 4–8 mice/group). Full details of all the sample sizes for each parameter analysed are provided in every figure legend.

### Oral gavage with fluorescent microbeads

Mice were given a single oral gavage of 2x10^11^ of Fluoresbrite Yellow Green labelled 200 nm microbeads (Polysciences, Eppelheim, Germany) in 200 μl PBS. Mice were culled 24 h later and Peyer’s patches and small intestine segments were snap-frozen at the temperature of liquid nitrogen. Serial frozen sections (6 μm in thickness) were cut on a cryostat and counterstained with DAPI. Images of SED from three Peyer’s Patches (duodenal, jejunal and ileal) and 8 LP areas per mouse (*n* = 3–4 mice/group) from 3 non-sequential sections (total 21–31 SED, or 24 LP areas per mouse studied) were typically acquired using a Nikon Eclipse E400 fluorescent microscope using Micro Manager (http://www.micro-manager.org). For example, each Peyer’s patch was trimmed until at least one SED region was visible and 20 sections collected. The 1^st^, 10^th^ and 20^th^ sections were then analysed. Tissue auto-fluorescence was subtracted from displayed images using ImageJ, the size of the area of interest in each section was then measured and the number of beads determined using the cell counter function in ImageJ and the bead density calculated.

### *In vitro* enteroid cultivation

Intestinal crypts were dissociated from mouse small intestine using Gentle Cell Dissociation Reagent (Stemcell Tech, Cambridge, UK) and used establish enteroids by cultivation in Matrigel (BD Bioscience) and Intesticult medium (Stemcell Tech) as described [23, 90]. Where indicated, some wells were treated with RANKL (100 ng/ml). Enteroids were cultivated in triplicate and either passaged after 5 d of cultivation [90] or harvested for mRNA expression analysis as described [23].

### Real-time quantitative PCR (RT-qPCR) analysis of mRNA expression

Total RNA was isolated from the enteroid cultures using RNA-Bee (AMS Biotechnology, Oxfordshire, UK) followed by treatment with DNase I (Ambion, Warrington, UK). First strand cDNA synthesis was performed using 1 μg of total RNA and the First Strand cDNA Synthesis kit (GE Healthcare, Bucks, UK) as described by the manufacturer. PCR was performed using the Platinum-SYBR Green qPCR SuperMix-UDG kit (Life Technologies) and the Stratagene Mx3000P real-time qPCR system (Stratagene, CA, USA). The qPCR primers (S1 Table) were designed using Primer3 software [91]. The cycle threshold values were determined using MxPro software (Stratagene) and normalized relative to *Gapdh*.

### Statistical analyses

All data are presented as mean ± SD. Unless indicated otherwise, differences between groups were analysed by a Student's t-test. In instances where there was evidence of non-normality (identified by the Kolmogorov-Smirnov, D’Agostino & Pearson omnibus, or Shapiro-Wilk normality tests), data were analysed by a Mann-Whitney *U* test. Survival rates were analysed using the Log-rank (Mantel-Cox) test. Values of *P*<0.05 were accepted as significant.

## Supporting Information

S1 Table Primers used for RT-qPCR analysis. (DOCX)

S1 Fig RANKL-treatment induces the expression of M cell-related genes in *in vitro* cultivated enteroids. Enteroids were prepared from the small intestines of RANK^F/F^ and RANK^ΔIEC^ mice. Following passage the enteroids were treated with either RANKL (100 ng) or PBS as a control. The expression of (A) M cell, (B) Paneth cell, and (C) intestinal stem cell-related genes was compared 7 d after treatment (*n* = 3 enteroid cultures/group). Gene expression was determined by RT-qPCR and normalized to the expression level of *Gapdh* (mean ± SD).
(TIF)

S2 Fig Effect of RANKL-treatment follicular dendritic cell (FDC) status. Immunohistochemical (IHC) and morphometric analyses were used to determine whether RANKL-treatment influenced the status of follicular dendritic cells (FDC) in Peyer’s patches and mesenteric lymph nodes (MLN). C57BL/6 mice were treated daily with RANKL (or PBS as a control) to induce M cell-differentiation, and Peyer’s patches, intestines and MLN collected on d 3. A) IHC comparison of CD21/35 (red) and PrP^C^ (blue) expression by FDC in the B cell-follicles (B220^+^ cells, green) of Peyer’s patches from RANKL- and PBS-treated mice. B) Morphometric analysis suggested that the area of the CD21/35^+^ immunostaining in the Peyer’s patches of mice from each treatment group was similar (*P* = 0.104, Student’s *t*-test; data derived from 3–4 follicles/mouse, *n* = 4 mice/group). C) Morphometric analysis suggested that the % area of PrP^C^ immunostaining within the CD21/35^+^ FDC networks was also similar in Peyer’s patches of mice from each treatment group (*P* = 0.485, Mann-Whitney *U* test; data derived from 2–8 follicles/mouse, *n* = 3 mice/group). D) Sections of MLN from RANKL- and PBS-treated mice were immunostained to detect B cells (B220, green), FDC (CD21/35^+^ cells, red) and PrP^C^ (blue). E) Morphometric analysis similarly suggested that the % area of PrP^C^ immunostaining within the FDC networks was equivalent in the MLN from RANKL- and PBS-treated mice (*P* = 0.065, Mann-Whitney *U* test; data derived from 2–6 follicles/mouse, *n* = 4 mice/group).
(TIF)

S3 Fig Prion accumulation in the lymphoid tissues of PBS- and RANKL-treated mice at the terminal stage of disease. C57BL/6 mice were treated daily for 4 d with RANKL (or PBS as a control) to induce M cell-differentiation, and orally-exposed to a limiting (0.1%) dose of ME7 scrapie prions between the 3^rd^ and 4^th^ treatments. Peyer’s patches, mesenteric lymph nodes (MLN) and spleen were collected from all clinically-affected mice and those which were free of the clinical signs of prion disease at the end of the experiment at 525 days post infection (dpi). Clin., clinical prion disease status; pos., clinically positive; neg. clinically negative; individual survival times are shown. High levels of PrP^Sc^ (PET immunoblot, black, arrows) were detected in association with follicular dendritic cells (CD21/35^+^ cells, brown, arrows) in the Peyer’s patches, MLN and spleens from all clinically-affected mice. In contrast, no PrP^Sc^ was detected in tissues from any of the clinically-negative survivors at 525 dpi. Sections were counterstained with haematoxylin to detect cell nuclei (blue). 0.1%-PBS Clin. pos, *n* = 3 mice; 0.1%-PBS Clin. neg, *n* = 5 mice; 0.1%-RANKL Clin. pos, *n* = 7 mice; 0.1%-RANKL Clin. neg, *n* = 1 mouse.
(TIF)

S4 Fig RANKL-treatment does not facilitate prion accumulation in the Peyer’s patches and mesenteric lymph nodes (MLN) of RANK^ΔIEC^ mice orally exposed to prions. RANK^ΔIEC^ mice were treated daily for 4 d with RANKL and orally-exposed to a 1% dose of ME7 scrapie prions between the 3^rd^ and 4^th^ treatments. Wild-type (WT) mice orally-exposed to prions alone were included as a control. At 105 days post-infection, heavy accumulations of PrP^Sc^ (PET immunoblot, black, arrows) in association with FDC (CD21/35^+^ cells, brown, arrows) were clearly evident in the Peyer’s patches and MLN of WT mice (left-hand panels). In contrast, no PrP^Sc^ accumulation was observed in tissues from the RANKL-treated RANK^ΔIEC^ mice orally exposed to prions (right-hand panels). Sections were counterstained with haematoxylin to detect cell nuclei (blue). Images are representative of tissues from 4 mice/group.
(TIF)

S5 Fig Primary antibody controls. Images of Peyer’s patches showing typical examples of the immunostaining obtained with the primary Ab used in this study (first and third columns) and their corresponding negative controls (second and fourth columns). Sections were counterstained with DAPI (blue) to detect cell nuclei. The antibody concentrations or dilutions used are indicated. All scale bars = 50 μm.
(TIF)
