# Correction: Increased Abundance of M Cells in the Gut Epithelium Dramatically Enhances Oral Prion Disease Susceptibility

**DOI:** 10.1371/journal.ppat.1006222

**Published:** 2017-02-17

**Authors:** The PLOS Pathogens Staff

The Data Availability statement for this paper is incorrect. The correct statement is: The authors confirm that all data underlying the findings are fully available without restriction. All the raw data presented within the study is available at DataShare and can be found at the following DOI: http://dx.doi.org/10.7488/ds/1563
