# Supplementary material for: Increased Abundance of M Cells in the Gut Epithelium Dramatically Enhances Oral Prion Disease Susceptibility
Source: PLoS Pathog. 2016 Dec 14;12(12):e1006075. doi: 10.1371/journal.ppat.1006075 (PMC5156364; doi:10.1371/journal.ppat.1006075)
Supplement: S1 Table — (DOCX) [file ppat.1006075.s001.docx]

**S1 Table:** Primers used for RT-qPCR analysis

| **Gene** | **Forward primer (5’ – 3’)** | **Reverse primer (5’ – 3’)** |
| --- | --- | --- |
| *Anxa5* | TTTCCGTTGCACGGAGTTGT | TTTCCTGGCGCTGAGCATT |
| *Ccl9* | TACTGCCCTCTCCTTCCTCA | TTGAAAGCCCATGTGAAACA |
| *Gapdh* | GATACTGCACAGACCCCTCCA | GCAGTTCCGGTCATTGAGGTA |
| *Gp2* | GATACTGCACAGACCCCTCCA | GCAGTTCCGGTCATTGAGGTA |
| *Lgr5* | GGGAGCGTTCACGGGCCTTC | GGTTGGCATCTAGGCGCAGGG |
| *Lyz1* | GAGACCGAAGCACCGACTATG | CGGTTTTGACATTGTGTTCGC |
| *Lyz2* | ATGGAATGGCTGGCTACTATGG | ACCAGTATCGGCTATTGATCTGA |
| *Marcksl1* | TTTTGCCCTCCTGTGGATTCT | CCACTAGGCACAGCACAAGAGA |
| *Muc1* | CCCTACCTACCACACACTCACGGACG | GTGGTCACCACAGCTGGGTTGGTA |
| *Muc2* | CTGACCAAGAGCGAACACAA | CATGACTGGAAGCAACTGGA |
| *SpiB* | AGCGCATGACGTATCAGAAGC | GGAATCCTATACACGGCACAGG |
