# Supplementary figures and images for: Increased Abundance of M Cells in the Gut Epithelium Dramatically Enhances Oral Prion Disease Susceptibility
Source: PLoS Pathog. 2016 Dec 14;12(12):e1006075. doi: 10.1371/journal.ppat.1006075 (PMC5156364; doi:10.1371/journal.ppat.1006075)

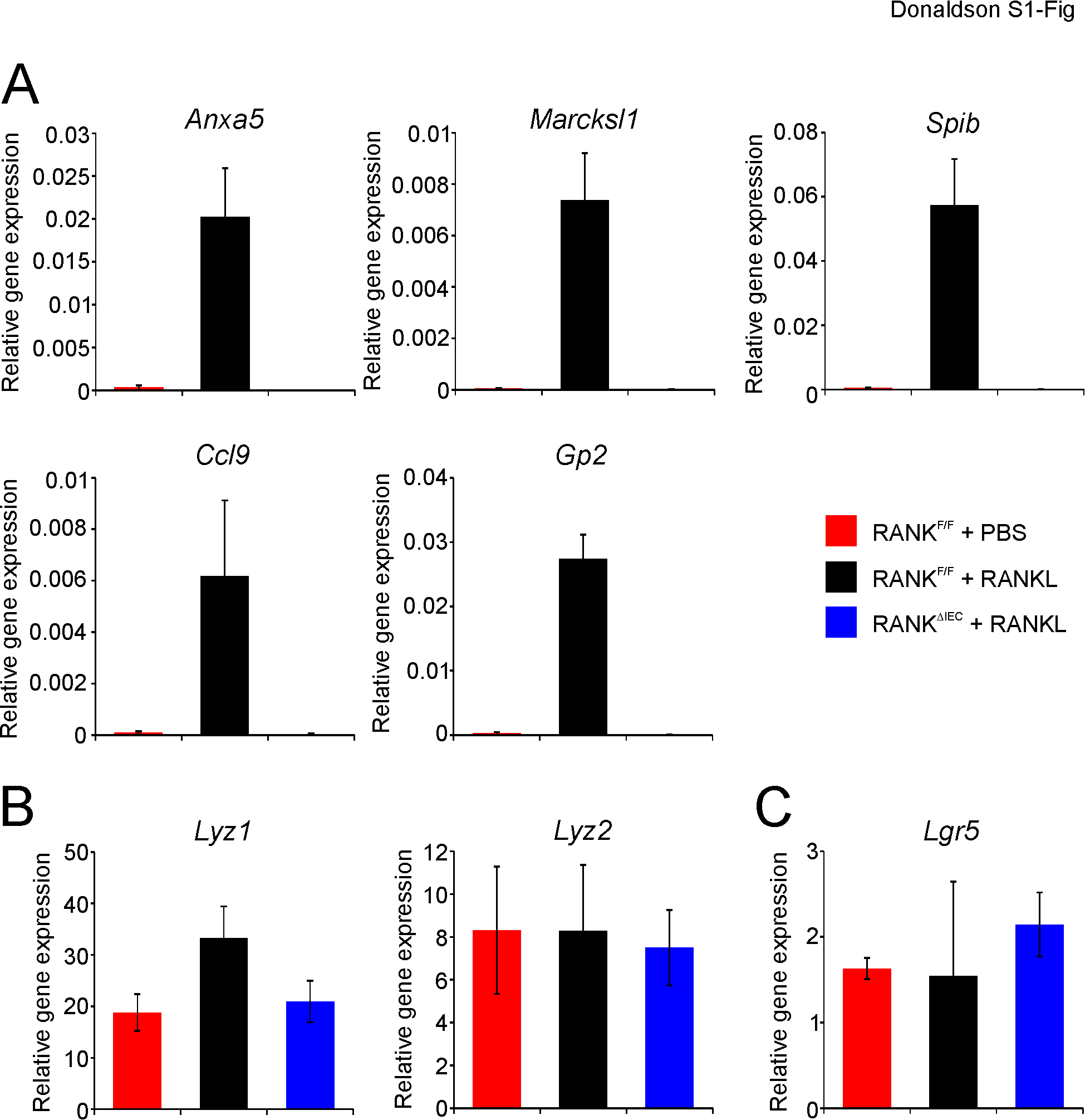

Supplement: S1 Fig — Enteroids were prepared from the small intestines of RANKF/F and RANKΔIEC mice. Following passage the enteroids were treated with either RANKL (100 ng) or PBS as a control. The expression of (A) M cell, (B) Paneth cell, and (C) intestinal stem cell-related genes was compared 7 d after treatment (n = 3 enteroid cultures/group). Gene expression was determined by RT-qPCR and normalized to the expression level of Gapdh (mean ± SD). (TIF) [file ppat.1006075.s002.tif]

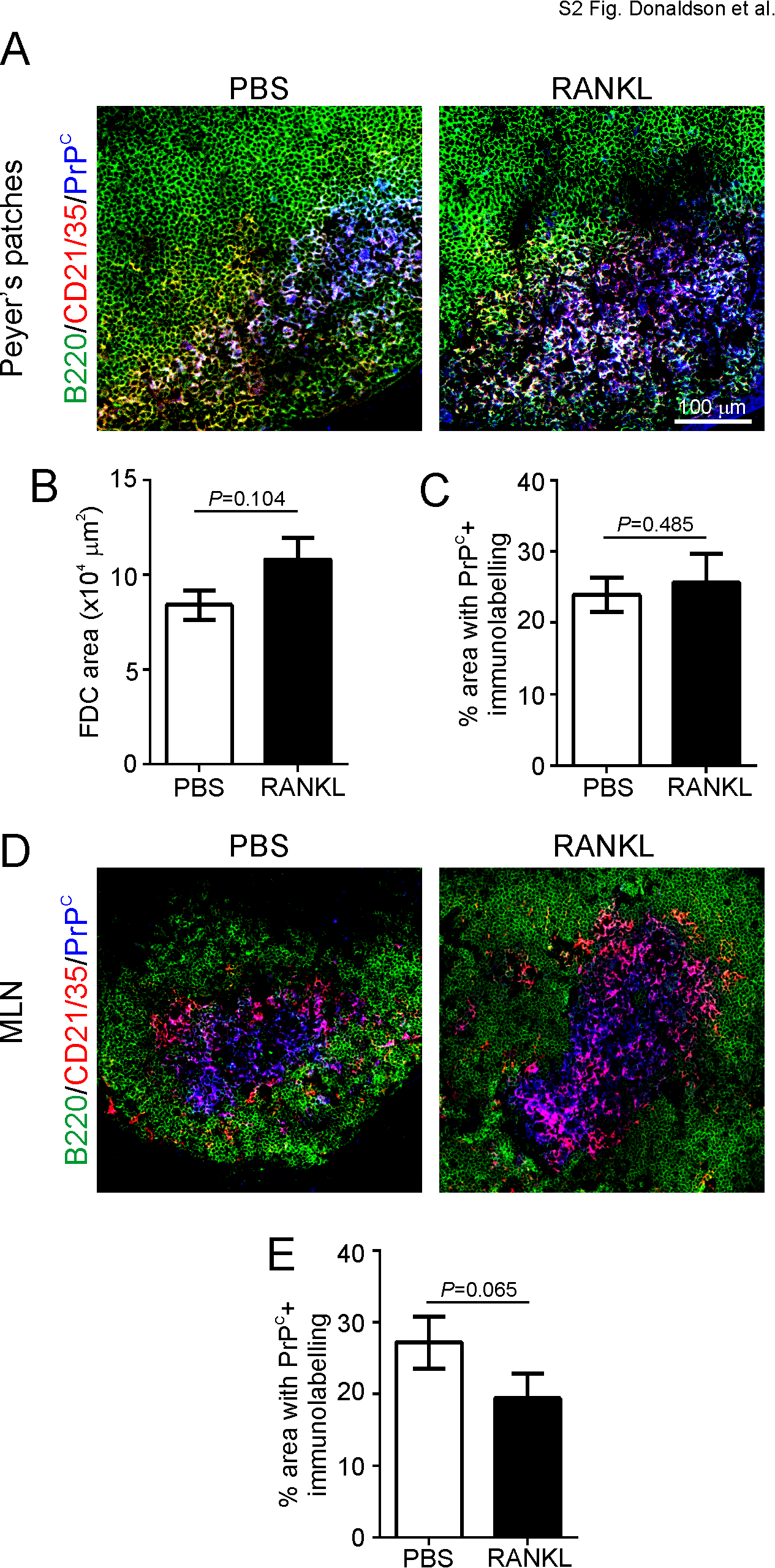

Supplement: S2 Fig — Immunohistochemical (IHC) and morphometric analyses were used to determine whether RANKL-treatment influenced the status of follicular dendritic cells (FDC) in Peyer’s patches and mesenteric lymph nodes (MLN). C57BL/6 mice were treated daily with RANKL (or PBS as a control) to induce M cell-differentiation, and Peyer’s patches, intestines and MLN collected on d 3. A) IHC comparison of CD21/35 (red) and PrPC (blue) expression by FDC in the B cell-follicles (B220+ cells, green) of Peyer’s patches from RANKL- and PBS-treated mice. B) Morphometric analysis suggested that the area of the CD21/35+ immunostaining in the Peyer’s patches of mice from each treatment group was similar (P = 0.104, Student’s t-test; data derived from 3–4 follicles/mouse, n = 4 mice/group). C) Morphometric analysis suggested that the % area of PrPC immunostaining within the CD21/35+ FDC networks was also similar in Peyer’s patches of mice from each treatment group (P = 0.485, Mann-Whitney U test; data derived from 2–8 follicles/mouse, n = 3 mice/group). D) Sections of MLN from RANKL- and PBS-treated mice were immunostained to detect B cells (B220, green), FDC (CD21/35+ cells, red) and PrPC (blue). E) Morphometric analysis similarly suggested that the % area of PrPC immunostaining within the FDC networks was equivalent in the MLN from RANKL- and PBS-treated mice (P = 0.065, Mann-Whitney U test; data derived from 2–6 follicles/mouse, n = 4 mice/group). (TIF) [file ppat.1006075.s003.tif]

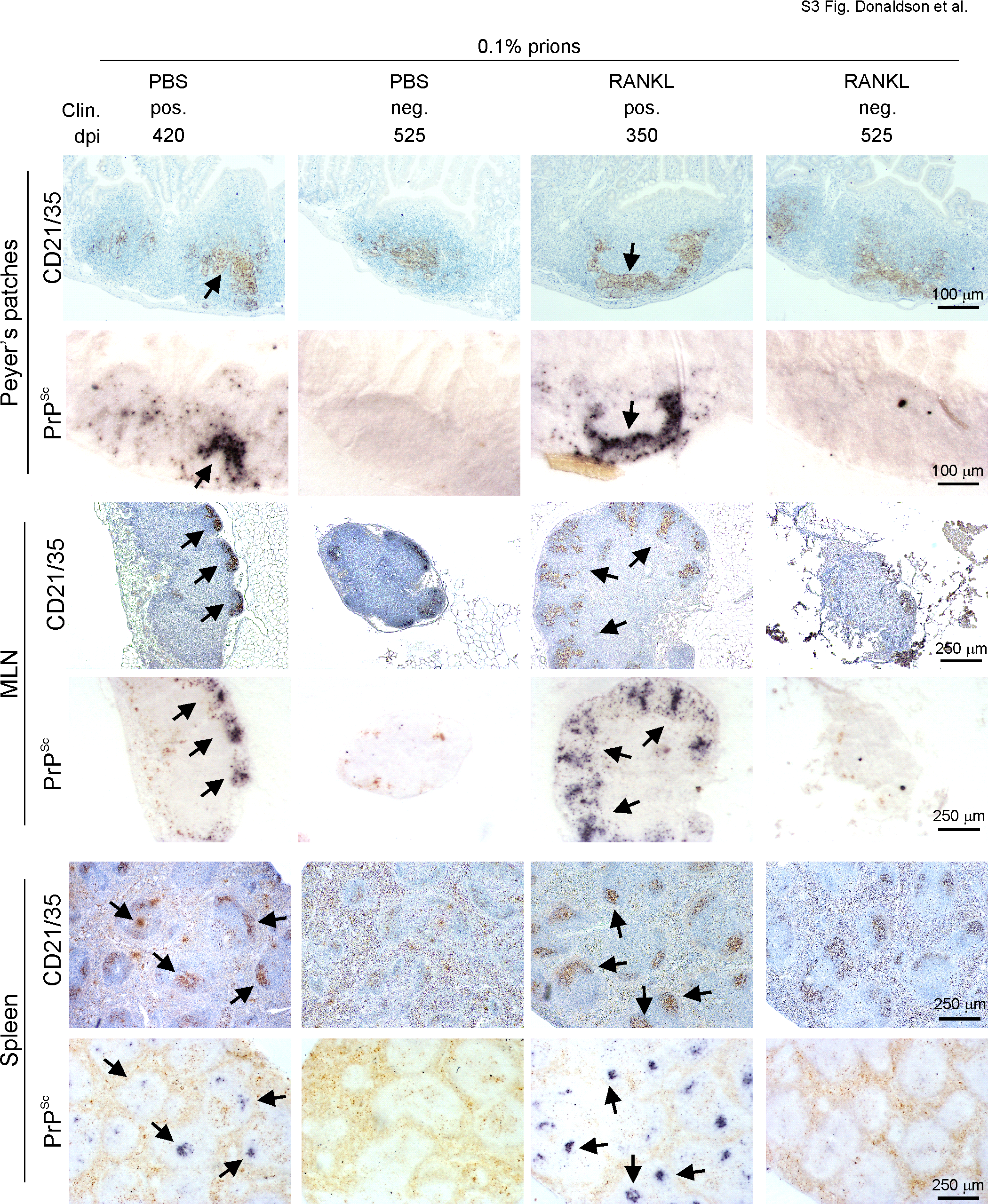

Supplement: S3 Fig — C57BL/6 mice were treated daily for 4 d with RANKL (or PBS as a control) to induce M cell-differentiation, and orally-exposed to a limiting (0.1%) dose of ME7 scrapie prions between the 3rd and 4th treatments. Peyer’s patches, mesenteric lymph nodes (MLN) and spleen were collected from all clinically-affected mice and those which were free of the clinical signs of prion disease at the end of the experiment at 525 days post infection (dpi). Clin., clinical prion disease status; pos., clinically positive; neg. clinically negative; individual survival times are shown. High levels of PrPSc (PET immunoblot, black, arrows) were detected in association with follicular dendritic cells (CD21/35+ cells, brown, arrows) in the Peyer’s patches, MLN and spleens from all clinically-affected mice. In contrast, no PrPSc was detected in tissues from any of the clinically-negative survivors at 525 dpi. Sections were counterstained with haematoxylin to detect cell nuclei (blue). 0.1%-PBS Clin. pos, n = 3 mice; 0.1%-PBS Clin. neg, n = 5 mice; 0.1%-RANKL Clin. pos, n = 7 mice; 0.1%-RANKL Clin. neg, n = 1 mouse. (TIF) [file ppat.1006075.s004.tif]

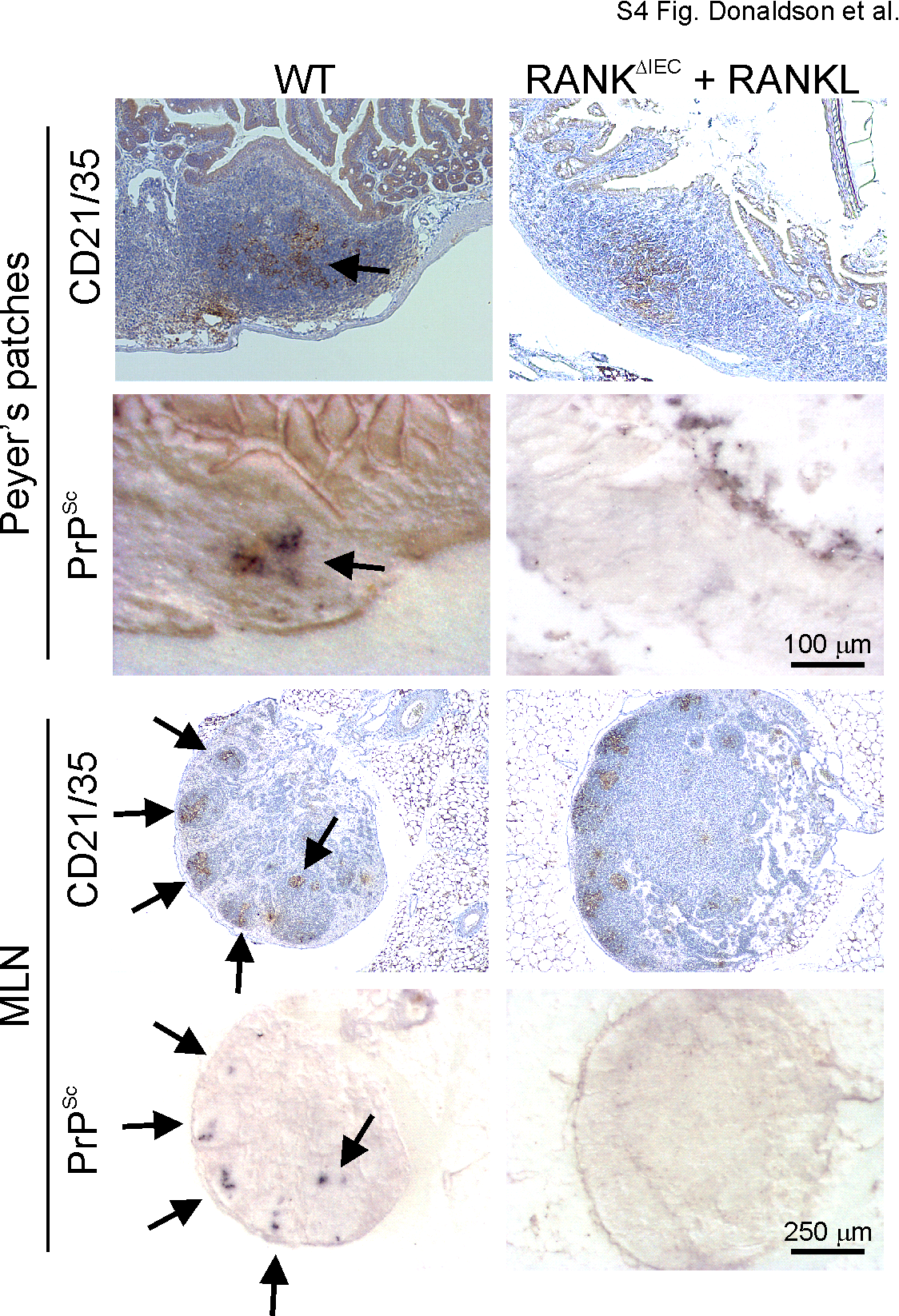

Supplement: S4 Fig — RANKΔIEC mice were treated daily for 4 d with RANKL and orally-exposed to a 1% dose of ME7 scrapie prions between the 3rd and 4th treatments. Wild-type (WT) mice orally-exposed to prions alone were included as a control. At 105 days post-infection, heavy accumulations of PrPSc (PET immunoblot, black, arrows) in association with FDC (CD21/35+ cells, brown, arrows) were clearly evident in the Peyer’s patches and MLN of WT mice (left-hand panels). In contrast, no PrPSc accumulation was observed in tissues from the RANKL-treated RANKΔIEC mice orally exposed to prions (right-hand panels). Sections were counterstained with haematoxylin to detect cell nuclei (blue). Images are representative of tissues from 4 mice/group. (TIF) [file ppat.1006075.s005.tif]

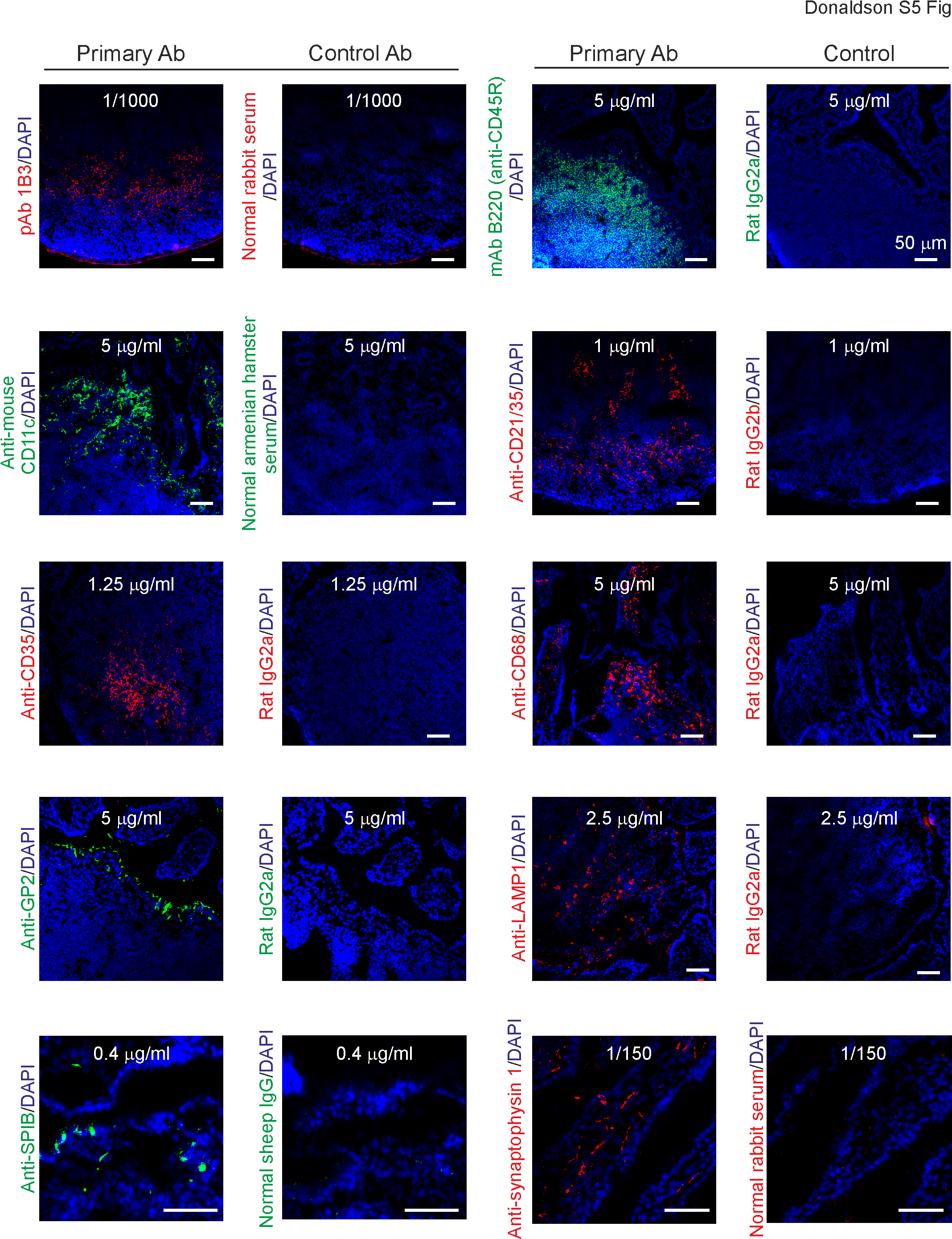

Supplement: S5 Fig — Images of Peyer’s patches showing typical examples of the immunostaining obtained with the primary Ab used in this study (first and third columns) and their corresponding negative controls (second and fourth columns). Sections were counterstained with DAPI (blue) to detect cell nuclei. The antibody concentrations or dilutions used are indicated. All scale bars = 50 μm. (TIF) [file ppat.1006075.s006.tif]
